# sTREM2 is associated with amyloid‐related p‐tau increases and glucose hypermetabolism in Alzheimer's disease

**DOI:** 10.15252/emmm.202216987

**Published:** 2023-01-09

**Authors:** Davina Biel, Marc Suárez‐Calvet, Paul Hager, Anna Rubinski, Anna Dewenter, Anna Steward, Sebastian Roemer, Michael Ewers, Christian Haass, Matthias Brendel, Nicolai Franzmeier, the Alzheimer's Disease Neuroimaging Initiative (ADNI)

**Affiliations:** ^1^ Institute for Stroke and Dementia Research (ISD), University Hospital LMU Munich Munich Germany; ^2^ Barcelonaβeta Brain Research Center (BBRC) Pasqual Maragall Foundation Barcelona Spain; ^3^ IMIM (Hospital del Mar Medical Research Institute) Barcelona Spain; ^4^ Servei de Neurologia Hospital del Mar Barcelona Spain; ^5^ Centro de Investigación Biomédica en Red de Fragilidad y Envejecimiento Saludable (CIBERFES) Madrid Spain; ^6^ Institute of Radiology and Artificial Intelligence and Informatics in Medicine TU Munich Munich Germany; ^7^ Department of Neurology, University Hospital LMU Munich Munich Germany; ^8^ German Center for Neurodegenerative Diseases (DZNE) Munich Germany; ^9^ Munich Cluster for Systems Neurology (SyNergy) Munich Germany; ^10^ Chair of Metabolic Biochemistry, Biomedical Center (BMC), Faculty of Medicine LMU Munich Munich Germany; ^11^ Department of Nuclear Medicine, University Hospital LMU Munich Munich Germany; ^12^ Department of Psychiatry and Neurochemistry, Institute of Neuroscience and Physiology, The Sahlgrenska Academy University of Gothenburg Gothenburg Germany

**Keywords:** Alzheimer's disease, beta‐amyloid, glucose metabolism, p‐tau, sTREM2, Biomarkers, Neuroscience

## Abstract

Microglial activation occurs early in Alzheimer's disease (AD) and previous studies reported both detrimental and protective effects of microglia on AD progression. Here, we used CSF sTREM2 to investigate disease stage‐dependent drivers of microglial activation and to determine downstream consequences on AD progression. We included 402 patients with measures of earliest beta‐amyloid (CSF Aβ_1‐42_) and late‐stage fibrillary Aβ pathology (amyloid‐PET centiloid), as well as sTREM2, p‐tau_181_, and FDG‐PET. To determine disease stage, we stratified participants into early Aβ‐accumulators (Aβ CSF+/PET−; *n* = 70) or late Aβ‐accumulators (Aβ CSF+/PET+; *n* = 201) plus 131 controls. In early Aβ‐accumulators, higher centiloid was associated with cross‐sectional/longitudinal sTREM2 and p‐tau_181_ increases. Further, higher sTREM2 mediated the association between centiloid and cross‐sectional/longitudinal p‐tau_181_ increases and higher sTREM2 was associated with FDG‐PET hypermetabolism. In late Aβ‐accumulators, we found no association between centiloid and sTREM2 but a cross‐sectional association between higher sTREM2, higher p‐tau_181_ and glucose hypometabolism. Our findings suggest that a TREM2‐related microglial response follows earliest Aβ fibrillization, manifests in inflammatory glucose hypermetabolism and may facilitate subsequent p‐tau_181_ increases in earliest AD.

## Graphical abstract

In Alzheimer's disease (AD), microglial activation has been linked to both detrimental and protective effects on disease progression. This study used CSF sTREM2 to investigate disease stage‐dependent drivers of microglial activation and to determine downstream consequences on AD progression.

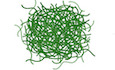

**The paper explained**

**Problem**

Besides beta‐amyloid (Aβ) and tau accumulation, microglial activation plays a role in the pathogenesis of Alzheimer's disease (AD). Previous studies reported both detrimental and protective effects of microglia on AD progression, thus, it is critical to investigate at which AD stages microglial activation could be protective or detrimental to evaluate microglia as a treatment target. To address this, we used CSF sTREM2 (i.e., soluble Triggering receptor expressed on myeloid cells 2) to investigate disease stage‐dependent drivers of microglial activation and to determine downstream consequences on AD biomarker progression.

**Results**

We first stratified groups into early and late Aβ‐accumulators to investigate disease stage‐dependent effects of CSF sTREM2 on AD progression. To that end, Aβ CSF positive but still amyloid‐PET negative participants were classified as early Aβ‐accumulators and participants that were both Aβ CSF and PET positive were classified as late Aβ‐accumulators. We found that in early Aβ‐accumulators, higher amyloid‐PET was associated with cross‐sectional/longitudinal CSF sTREM2 and CSF p‐tau_181_ increases, suggesting reactive microglial and p‐tau increases in response to earliest Aβ fibrillization. Further, higher CSF sTREM2 mediated the association between amyloid‐PET and cross‐sectional/longitudinal CSF p‐tau_181_ increases and higher CSF sTREM2 was associated with FDG‐PET hypermetabolism in line with previous findings of increased glucose consumption of activated microglia. In late Aβ‐accumulators, we found no association between amyloid‐PET and CSF sTREM2 but a cross‐sectional association between higher CSF sTREM2, higher CSF p‐tau_181_ and glucose hypometabolism, suggesting that sTREM2 parallels tau and neurodegeneration rather than Aβ once fully developed Aβ pathology is present.

**Impact**

Our findings suggest that sTREM2‐related microglial activation occurs in response to earliest Aβ fibrillization, manifests in inflammatory glucose hypermetabolism and may facilitate subsequent p‐tau increases in earliest AD, while previous reports of protective sTREM2 effects may occur in later AD stages.

## Introduction

Alzheimer's disease (AD) is characterized by the accumulation of beta‐amyloid (Aβ), tau, microglial activation, metabolic brain changes, neurodegeneration, and cognitive decline (Jack *et al*, 2018). According to the amyloid cascade hypothesis, Aβ accumulation triggers the subsequent development and spreading of hyperphosphorylated tau aggregates which is followed by neurodegeneration, metabolic decline, and eventually dementia (Karran *et al*, 2011; La Joie *et al*, 2020; Haass & Selkoe, 2022; Strom *et al*, 2022). Previously it was shown that Aβ‐related increases in soluble hyperphosphorylated tau (i.e., p‐tau), detectable in plasma (Janelidze *et al*, 2021; Moscoso *et al*, 2021) and cerebrospinal fluid (CSF) (Mattsson‐Carlgren *et al*, 2020), precede tau aggregation. We have shown recently that soluble p‐tau increases may in fact drive tau aggregation and spread across interconnected brain regions (Pichet Binette *et al*, 2022) therefore, soluble p‐tau increases may be a key link between Aβ deposition and tau aggregation in AD. However, the underlying mechanisms that link Aβ and subsequent increases in soluble p‐tau in CSF or plasma are not well understood.

Here, activation of microglia, the brains innate immune system, may play a key role in modulating these initial events in the amyloid cascade (Pascoal *et al*, 2021). The Triggering Receptor Expressed on Myeloid Cell 2 (TREM2) regulates the change of microglia from a homeostatic state to a disease associated state (Keren‐Shaul *et al*, 2017; Krasemann *et al*, 2017) and is a well‐established *in vivo* proxy for microglial activation in AD (Suarez‐Calvet *et al*, 2016, 2019; Ewers *et al*, 2019, 2020; Franzmeier *et al*, 2020). Yet, previous studies have yielded conflicting findings on a detrimental or protective role of microglial activation or TREM2‐related microglial responses in AD. For instance, recent *in vitro* studies reported that activated microglia can induce tau hyperphosphorylation and spread (Maphis *et al*, 2015) and that activated microglia can release tau seeds which can induce tau aggregation (Brelstaff *et al*, 2021). Similarly, studies in sporadic AD patients found that a TREM2‐related microglial response is strongly correlated with soluble p‐tau but not with Aβ levels (Suárez‐Calvet *et al*, 2019) and that a TREM2‐related microglial response may promote the development of aggregated tau pathology in AD, as measured via tau‐PET (Vogels *et al*, 2019; Pascoal *et al*, 2021). Indeed, a recent post‐mortem study investigating the mediating effect of microglial activation on the Aβ to tau association in brain tissue revealed a mediation effect of 33% of microglia for the relationship between Aβ and tau (Casaletto *et al*, 2022). This suggests that microglial activation may be associated with tau hyperphosphorylation and therefore contribute to the development of tau pathology in AD. In addition, activated microglia have been shown to consume high levels of glucose in AD mouse models and AD patients (Xiang *et al*, 2021), which may manifest in hypermetabolic brain changes that are observed in early‐stage AD, when neurodegeneration and ensuing glucose hypometabolism are not yet apparent (Oh *et al*, 2016; Gordon *et al*, 2018). Thus, glucose hypermetabolism in early AD may not reflect a compensatory mechanism, as suggested previously, but rather reflect activated microglia and neuroinflammation (Ashraf *et al*, 2015; Arenaza‐Urquijo *et al*, 2017).

On the contrary, in symptomatic sporadic AD patients and patients with autosomal dominantly inherited AD, a higher TREM2‐related microglial response has been associated with attenuated cognitive decline, amyloid accumulation and neurodegeneration (Ewers *et al*, 2019, 2020; Morenas‐Rodriguez *et al*, 2022). This suggests a possible protective effect of chronic microglial activation on neuronal integrity and cognition that occurs once pathologic brain changes are severe enough to result in clinically manifested cognitive deficits. Similarly, an animal model has shown that TREM2 loss of function is associated with increased Aβ seeding, further suggesting a protective effect of microglia on Aβ pathology development (Parhizkar *et al*, 2019). It is therefore of utmost importance for clinical trials trying to target microglial activation as a disease modifying approach to understand (i) what drives microglial activation in AD, (ii) whether and when microglial activation is beneficial or detrimental and (iii) whether the directionality of microglial effects on AD progression depends on disease stage.

In the present study, we used CSF soluble TREM2 (sTREM2) as an *in vivo* marker of TREM2‐related microglial responses in a well‐characterized sample of AD patients and controls to investigate drivers of microglial activation across early versus late‐stage Aβ accumulation in AD and its effects on the development of downstream p‐tau and metabolic brain changes. Specifically, we included data of 402 cognitively normal (CN) and mild cognitive impaired (MCI) participants from the ADNI database with available CSF Aβ_1‐42_, p‐tau_181_, and sTREM2, as well as amyloid‐PET, and ^18^F‐fluorodeoxyglucose PET (FDG‐PET). FDG‐PET is well established for assessing cerebral glucose uptake and FDG‐PET‐assessed hypermetabolism has been previously linked to microglial activation (Xiang *et al*, 2021). To determine disease stage, we classified patients into Aβ CSF+/PET− (early Aβ‐accumulators) and Aβ CSF+/PET+ (late Aβ‐accumulators) following a previously established approach that allows stratifying individuals by showing earliest signs of Aβ accumulation (i.e., Aβ CSF+/PET−) versus showing fully developed amyloid pathology (Aβ CSF+/PET+) (Palmqvist *et al*, 2017). A total of 131 participants without evidence of Aβ pathology were included as healthy controls (Aβ CSF−/PET−). A subset of participants had available longitudinal p‐tau_181_ and sTREM2 assessments, based on which we calculated annual sTREM2 and p‐tau_181_ change rates. Our specific aims were to assess first, whether earliest signs of Aβ accumulation (i.e., in Aβ CSF+/PET−) are associated with a TREM2‐related microglial response and second, whether this initial Aβ‐driven microglial activation facilitates subsequent increases in soluble hyperphosphorylated tau (i.e., p‐tau_181_). Third, we assessed whether earliest TREM2‐related microglial responses are reflected in increased FDG‐PET‐assessed glucose metabolism, given that activated microglia consume large amounts of glucose (Xiang *et al*, 2021). Here, we expected a higher TREM2‐related microglial response to be associated with glucose hypermetabolism in patients with earliest Aβ accumulation, where neurodegeneration is not yet apparent, versus hypometabolism in chronic AD phases within late Aβ‐accumulators.

## Results

We stratified participants by evidence for early‐ versus late‐stage Aβ pathology, using a previously established approach that combines CSF assessments of soluble Aβ_1‐42_ and amyloid‐PET assessments of fibrillar Aβ (Palmqvist *et al*, 2017). CSF Aβ abnormality is assumed to reflect early Aβ dysmetabolism and precedes amyloid‐PET positivity reflecting mostly fibrillary forms of Aβ (Palmqvist *et al*, 2016). Therefore, we grouped participants into (i) early Aβ‐accumulators (i.e., Aβ CSF+/PET−; *n* = 70; CN = 30; MCI = 40) with evidence for reduced Aβ_1‐42_ in CSF but no suprathreshold fibrillar Aβ pathology on PET versus (ii) late Aβ‐accumulators (i.e., Aβ CSF+/PET+; *n* = 201; CN = 41; MCI = 160), with evidence for abnormal Aβ in both CSF and PET. An additional pool of 131 cognitively normal subjects without evidence of abnormal Aβ on either CSF or PET was included as a control group. Longitudinal CSF data were available for a subset of participants for sTREM2 (early Aβ/late Aβ/controls *n* = 21/75/35) and p‐tau_181_ (early Aβ/late Aβ/controls *n* = 20/75/35) with an average follow‐up time from baseline CSF assessment of 1.99 ± 0.09 years. Descriptive baseline statistics stratified by groups are shown in Table 1.

**Table 1:** Demographic and clinical data stratified by group.

	Controls (Aβ CSF−/PET−)	Early Aβ‐accumulators (Aβ CSF+/PET−)	Late Aβ‐accumulators (Aβ CSF+/PET+)	*P*‐value
Cross‐sectional				
*N*	131	70	201	
Diagnostic (CN/MCI)	131/0	30/40	41/160	< 0.001
Sex (male/female)	64/67	47/23	112/89	0.045
Age in years	72.67 (6.39)	71.65 (7.68)	73.60 (6.53)	0.093
Years of education	16.85 (2.43)^a^	17.13 (2.24)^a^	16.00 (2.74)^b^ ^,^ ^c^	< 0.001
CSF Aβ_1‐42_ (pg/ml)	1,553.122 (281.109)^a^ ^,^ ^c^	777.819 (147.169)^a^ ^,^ ^b^	656.42 (174.79)^b^ ^,^ ^c^	< 0.001
CSF p‐tau_181_ (pg/ml)	20.241 (7.059)^a^ ^,^ ^c^	15.825 (7.493)^a^ ^,^ ^b^	32.807 (14.780)^b^ ^,^ ^c^	< 0.001
CSF sTREM2 (pg/ml)	4,099.933 (1,980.197)^c^	2,955.118 (1,751.746)^a^ ^,^ ^b^	3,984.107 (2,194.033)^c^	< 0.001
Amyloid‐PET (centiloid)	−9.0315 (12.656)^a^	−2.901 (13.530)^a^	78.228 (33.871)^b^ ^,^ ^c^	< 0.001
FDG‐PET global *z*‐score	‐	−0.132 (0.649)	−0.251 (0.532)	0.13
FDG‐PET meta‐ROI *z*‐score	‐	−0.214 (0.826)^a^	−0.546 (0.723)^c^	0.002
Longitudinal				
*N*	35	20	75	
Follow‐up CSF p‐tau_181_ (mean years)	1.96 (0.13)^c^	2.05 (0.08)^a^ ^,^ ^b^	1.99 (0.07)^c^	0.002
*N*	35	21	75	
Follow‐up CSF sTREM2 (mean years)	1.96 (0.13)^c^	2.05 (0.08)^b^	1.99 (0.07)	0.004

Values are presented as mean (SD); *P*‐values were derived from ANOVAs for continuous measures and from Chi‐squared tests for categorical measures. Mean values significantly (*P* < 0.05, *post hoc* tests) different from—.

^a^ Controls.

^b^ Early Aβ‐accumulators.

^c^ Late Aβ‐accumulators.

### Early but not late‐stage Aβ accumulation is associated with higher CSF sTREM2

**Figure 1:**
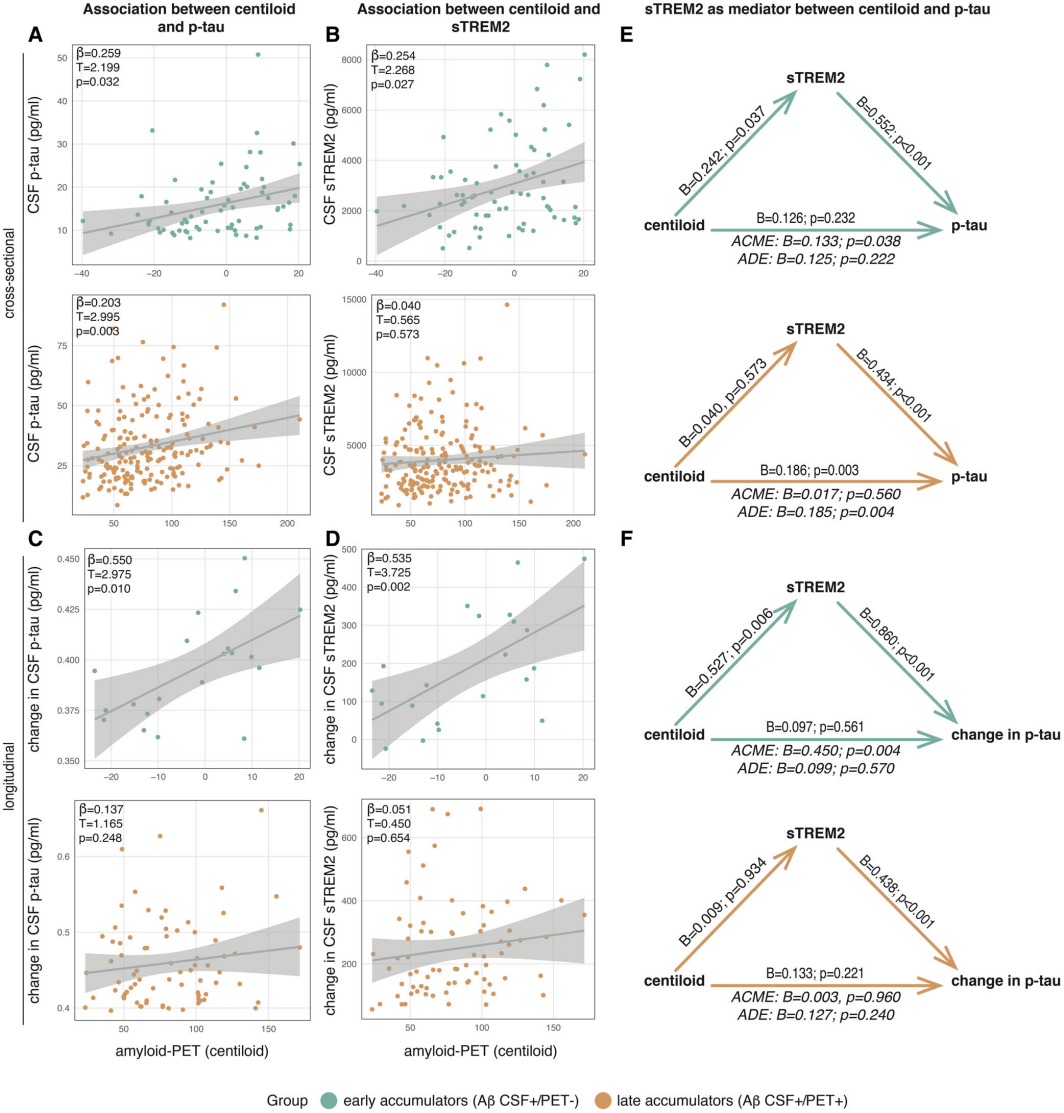
Cross‐sectional and longitudinal analysis of the association between amyloid‐PET (in centiloid), CSF p‐tau_181_, and CSF sTREM2 in early Aβ‐accumulators (i.e., Aβ CSF+/PET−; *n* = 70) and late Aβ‐accumulators (i.e., Aβ CSF+/PET+; *n* = 201) A–DCross‐sectional linear regressions between centiloid and p‐tau_181_ (A) and centiloid and sTREM2 (B). Longitudinal linear regressions between centiloid and change in p‐tau_181_ (C) and centiloid and change in sTREM2 (D). Standardized beta‐estimates (β), *T*‐values, and *P*‐values were derived from linear regressions.E–FCross‐sectional mediation analyses with centiloid as predictor, sTREM2 as mediator, and p‐tau_181_ as dependent variable (E). Longitudinal mediation analyses with centiloid as predictor, sTREM2 as mediator, and change in p‐tau_181_ as dependent variable (F). Beta‐estimates (*B*) and *P*‐values for each path are displayed on the respective arrow. The average causal mediation effect (ACME) and the average direct effect (ADE) are displayed under each mediation triangle. Data information: Early Aβ‐accumulators are displayed in green, and late Aβ‐accumulators in orange. All models are controlled for age, sex, education, and clinical status. Source data are available online for this figure.

We tested first whether evidence for earliest Aβ abnormality in CSF but not yet in PET (i.e., early Aβ accumulators) is associated with a sTREM2‐related microglial response and p‐tau_181_ increases. To this end, we used linear regression to determine the association between amyloid‐PET as a marker of fibrillary Aβ pathology (i.e., centiloid) and sTREM2 in early Aβ‐accumulators. Here, higher centiloid at baseline was associated with higher cross‐sectional p‐tau_181_ (β = 0.259, *T* = 2.199, *P* = 0.032; Fig 1A, top panel) and higher sTREM2 (β = 0.254, *T* = 2.268, *P* = 0.027; Fig 1B, top panel). Further, higher sTREM2 levels were associated with higher p‐tau_181_ levels (β = 0.587, *T* = 5.425, *P* < 0.001). We obtained congruent results using longitudinal CSF data, showing that higher centiloid at baseline was associated with faster subsequent change rates in p‐tau_181_ (β = 0.550, *T* = 2.975, *P* = 0.010; Fig 1C, top panel) and faster changes in sTREM2 (β = 0.535, *T* = 3.725, *P* = 0.002; Fig 1D, top panel). In addition, higher baseline sTREM2 levels were associated with faster subsequent change rates in p‐tau_181_ (β = 0.938, *T* = 6.286, *P* < 0.001). Using bootstrapped mediation analyses with 1,000 iterations, we additionally found that the association between higher centiloid and higher p‐tau_181_ was fully mediated by sTREM2 in early Aβ accumulators, both for cross‐sectional p‐tau_181_ (average causal mediation effect [ACME]: *B* = 0.133, 95% CI = 0.0039 to 0.27, *P* = 0.038; Fig 1E, top panel) as well as subsequent p‐tau_181_ change rates (ACME: *B* = 0.450, 95% CI = 0.1352 to 0.82, *P* = 0.004; Fig 1F, top panel). Testing the reverse mediation models, i.e., whether p‐tau_181_ mediates the effect of centiloid on sTREM2 yielded a similar mediation effect for cross‐sectional sTREM2 levels (ACME: *B* = 0.132, CI = 0.0084 to 0.26, *P* = 0.030) but a much lower mediation effect for subsequent sTREM2 change rates in longitudinal analyses (ACME: *B* = 0.273, CI = 0.0437 to 0.55, *P* = 0.014). These results suggest that earliest Aβ accumulation may induce a reactive TREM2‐related microglial response which may in turn facilitate p‐tau_181_ increases.

When assessing the above‐described analyses in late Aβ‐accumulators, no association was found between centiloid and sTREM2, neither for cross‐sectional sTREM2 (β = 0.040, *T* = 0.565, *P* = 0.573; Fig 1B, bottom panel) nor for longitudinal sTREM2 change rates (β = 0.051, *T* = 0.450, *P* = 0.654; Fig 1D, bottom panel). This suggests that sTREM2 increases are no longer driven by Aβ once fully developed fibrillar Aβ pathology is present. Yet, there was an association between higher baseline sTREM2, higher baseline p‐tau_181_ (β = 0.441, *T* = 7.004, *P* < 0.001) and longitudinal changes in p‐tau_181_ (β = 0.439, *T* = 3.777, *P* < 0.001), suggesting that sTREM2 is more strongly coupled to p‐tau_181_ in late Aβ‐accumulators. Higher centiloid was associated with higher cross‐sectional p‐tau_181_ (β = 0.203, *T* = 2.995, *P* = 0.003; Fig 1A, bottom panel), but no association was found between baseline centiloid and subsequent p‐tau_181_ change rates (*n* = 75, β = 0.137, *T* = 1.165, *P* = 0.248; Fig 1C, bottom panel). Given that there was no association between centiloid and sTREM2 in late Aβ‐accumulators, we did not detect a mediation effect of sTREM2 for the association between centiloid and p‐tau_181_, neither cross‐sectionally (ACME: *B* = 0.017, CI = −0.0444 to 0.08, *P* = 0.560; Fig 1E, bottom panel), nor longitudinally (ACME: *B* = 0.003, CI = −0.108 to 0.10, *P* = 0.960; Fig 1F, bottom panel).

Together, these findings suggest that earliest fibrillization of Aβ is associated with reactive sTREM2 increases, which may in turn precede increases in p‐tau_181_ in early Aβ‐accumulators, while sTREM2 dynamics may uncouple from the extent of fibrillar Aβ pathology at later stages but rather parallel p‐tau_181_ increases.

All analyses remained consistent when including APOE4 genotype as a covariate (except for the cross‐sectional ACME in early Aβ‐accumulators, which only reached borderline significance (*P* = 0.058; Tables EV1 and EV2)).

No sTREM2 by group interaction on CSF p‐tau_181_ levels were found when pooling early and late Aβ‐accumulators (cross‐sectional interaction term: β = 0.022, *T* = 0.145, *P* = 0.885; longitudinal interaction term: β = −0.091, *T* = −0.345, *P* = 0.731).

When performing the same analyses in Aβ CSF−/PET− healthy controls, no significant cross‐sectional or longitudinal associations between centiloid and sTREM2 (cross‐sectional: β = 0.118, *T* = 1.273, *P* = 0.205; longitudinal: β = −0.049, *T* = −0.240, *P* = 0.812) or p‐tau_181_ (cross‐sectional: β = 0.091, *T* = 0.967, *P* = 0.335; longitudinal: β = −0.119, *T* = −0.582, *P* = 0.565) were observed, suggesting that the association between Aβ fibrillization and sTREM2 increases are specific for patients who show earliest signs of AD pathophysiology as indicated by reduced CSF Aβ_1‐42_ levels.

Lastly, the above‐described analyses were repeated using CSF Aβ_1‐42_ instead of centiloid for testing associations between soluble Aβ and p‐tau_181_ or sTREM2. Except for an association between CSF Aβ_1‐42_ and p‐tau_181_ in late accumulators (*P* = 0.004), no associations were observed (Table 2), which is in accordance with previous work (Suarez‐Calvet *et al*, 2019). This suggests that sTREM2 increases are specifically associated with fibrillization of Aβ as assessed via amyloid‐PET.

**Table 2:** Associations between CSF Aβ_1‐42_ and p‐tau_181_ or sTREM2.

	Cross‐sectional			Longitudinal		
	β	*T*	*P*	β	*T*	*P*
Early Aβ‐accumulators (Aβ CSF+/PET−)						
p‐tau_181_ ~ Aβ_1‐42_	−0.225	−1.911	0.061	−0.215	−0.863	0.403
sTREM2 ~ Aβ_1‐42_	0.128	1.124	0.265	−0.006	−0.029	0.978
Late Aβ‐accumulators (Aβ CSF+/PET+)						
p‐tau_181_ ~ Aβ_1‐42_	0.196	2.894	0.004	0.094	0.791	0.432
sTREM2 ~ Aβ_1‐42_	0.069	0.979	0.329	0.134	1.197	0.236

The table displays standardized beta‐estimates (β), *T*‐values, and *P*‐values. The regression models are controlled for age, sex, education, and clinical status.

### Microglial activation is reflected in cerebral glucose metabolism across AD stages

**Figure 2:**
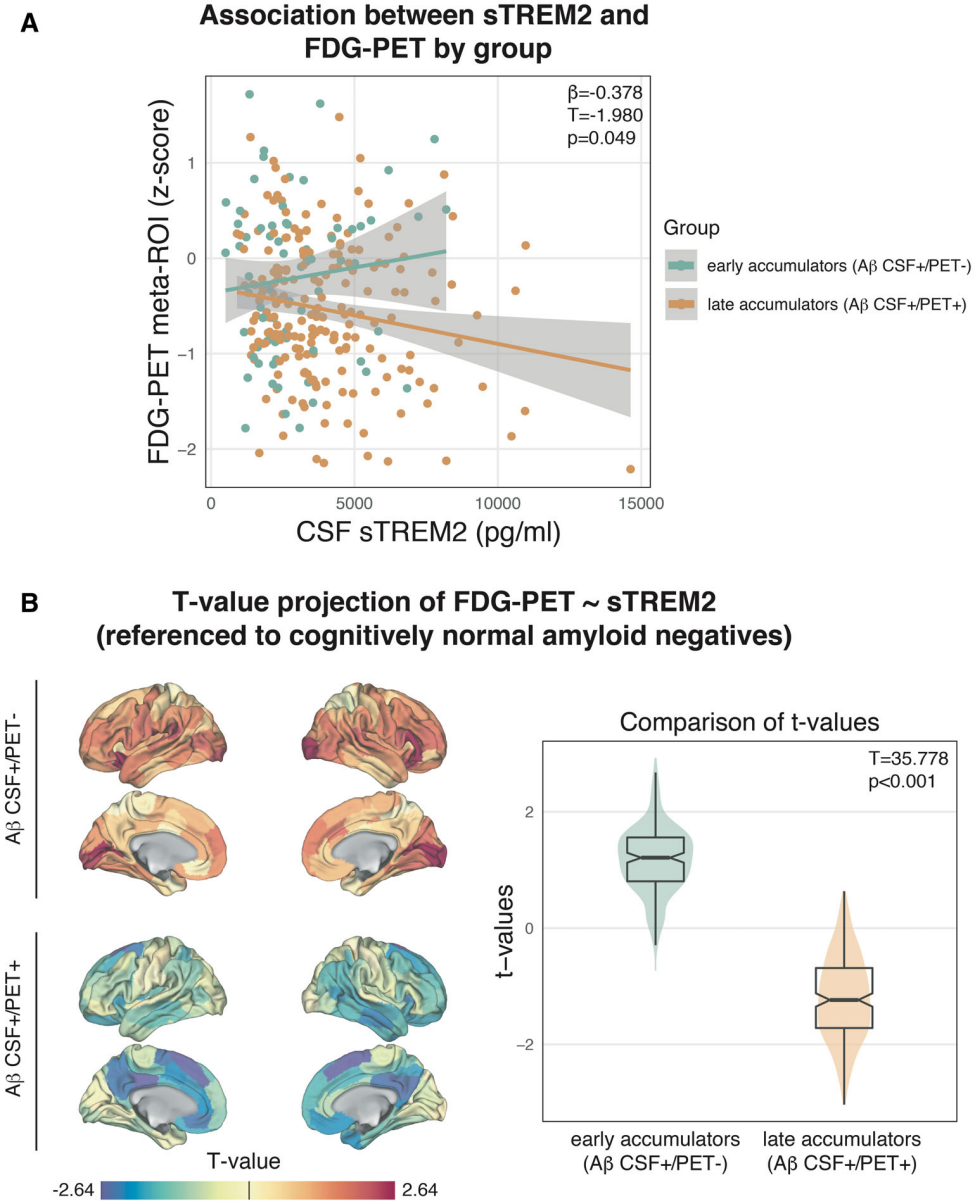
Association between CSF sTREM2 and FDG‐PET in early Aβ‐accumulators (i.e., Aβ CSF+/PET−; *n* = 70) and late Aβ‐accumulators (i.e., Aβ CSF+/PET+; *n* = 201) Plot shows significant (*P* < 0.05) sTREM2 by group interaction on FDG‐PET *z*‐scores within an FDG‐PET meta‐ROI (Landau *et al*, 2011) using a linear regression.
*T*‐value projection of the association between sTREM2 on FDG‐PET, stratified by group. Data information: FDG‐PET *z*‐scores were derived by referencing FDG‐PET SUVRs to cognitively normal controls (i.e., *n* = 131; Aβ CSF−/PET−). The models are controlled for age, sex, education, and clinical status. Boxplots are displayed as median (center line) ± inter‐quartile range (box boundaries) with whiskers including observations falling within the 1.5 interquartile range. 200 Regions of interest are displayed per group. Source data are available online for this figure.

**Figure EV1:**
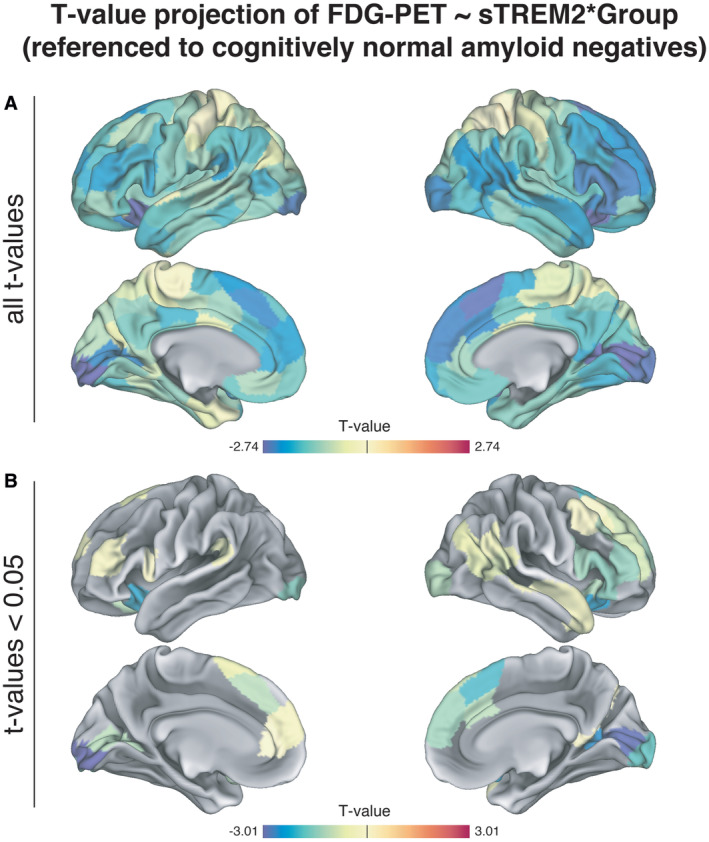
*T*‐value projection of the CSF sTREM2 by group (early Aβ‐accumulators [Aβ CSF+/PET−] vs. late Aβ‐accumulators [Aβ CSF+/PET+]) interaction on FDG‐PET Projection of all *T*‐values across 200 ROIs of the Schaefer brain atlas.Projection of *T*‐values that are below a *P*‐value of 0.05. Data information: FDG‐PET *z*‐scores were derived by referencing FDG‐PET SUVRs to cognitively normal controls (i.e., *n* = 131; Aβ CSF−/PET−). The models are controlled for age, sex, education, and clinical status.

Next, we tested whether there is a non‐linear relationship between sTREM2 levels and FDG‐PET‐assessed glucose metabolism across the spectrum of Aβ accumulation compared to controls. Microglia consume large amounts of glucose (Xiang *et al*, 2021), hence a TREM2‐related microglial response may result in FDG‐PET hypermetabolism in the earliest stages of Aβ accumulation, where neurodegeneration is typically not yet apparent. In late Aβ accumulators, a TREM2‐related microglial response parallels p‐tau_181_ which has been associated with subsequent neurodegeneration (Ossenkoppele *et al*, 2021; Pichet Binette *et al*, 2022). Hence higher sTREM2 may be associated with FDG‐PET hypometabolism in subjects with late‐stage Aβ accumulation. To test this, we summarized FDG‐PET of early and late Aβ‐accumulators across AD vulnerable brain regions (Landau *et al*, 2011) and referenced the mean FDG‐PET signal to 131 controls (i.e., Aβ CSF−/PET−) to derive FDG‐PET *z*‐scores that allow to determine whether FDG‐PET metabolism is higher or lower than in a reference group of healthy controls. Using linear regression, we observed a significant sTREM2 by group interaction on FDG‐PET meta‐ROI *z*‐scores (β = −0.378, *T* = −1.980, *P* = 0.049; Fig 2A, controlled for age, sex, education, and clinical status), showing that sTREM2 was associated with higher FDG‐PET in early Aβ‐accumulators versus lower FDG‐PET in late Aβ‐accumulators. The analyses remained consistent when including APOE4 genotype as a covariate (β = −0.379, *T* = −1.972, *P* = 0.050). To exploratory map the spatial pattern of brain regions in which higher sTREM2 is associated with higher FDG‐PET in early Aβ‐accumulators, versus lower FDG‐PET uptake in late Aβ‐accumulators, we assessed the association between sTREM2 and FDG‐PET *z*‐scores for early and late Aβ‐accumulators separately across 200 cortical brain regions included in the Schaefer brain atlas (Schaefer *et al*, 2018) using linear regression models controlling for age, sex, education, and clinical status. When projecting the *T*‐values of the association between sTREM2 levels and FDG‐PET to the brain surface (Fig 2B, left panel), we found consistent and brain‐wide positive associations between sTREM2 and FDG‐PET for early Aβ‐accumulators, versus consistent negative associations between sTREM2 and FDG‐PET for late Aβ‐accumulators. Comparing the *T*‐value distributions of the association between sTREM2 levels and FDG‐PET between early and late Aβ‐accumulators showed a significant group difference (*T* = 35.78, *P* < 0.001, Fig 2B, right panel). In addition, 95% CIs of the 200 *T*‐values did not overlap (early Aβ‐accumulators: CI 1.086 to 1.249; late Aβ‐accumulators: CI −1.302 to −1.098), supporting the view that there is a non‐linear relationship between sTREM2 levels and FDG‐PET across the spectrum of Aβ deposition, where sTREM2 is associated with relative FDG‐PET hypermetabolism in early Aβ‐accumulators versus relative hypometabolism in late Aβ‐accumulators. The brain surface projection of the *T*‐values of the sTREM2 by group interaction on FDG‐PET is made available in the Expanded View (Fig EV1). These findings suggest that elevated glucose metabolism in the early disease stage, before neurodegeneration is present, reflects neuroinflammation while in the later disease stage, higher sTREM2 parallels neuronal loss and tau aggregation, and therefore may manifest in metabolic decline.

## Discussion

In this combined CSF biomarker and neuroimaging study, we systematically assessed the correlates of earliest sTREM2‐related microglial responses in AD and its consequences on downstream changes in the amyloid cascade, including soluble p‐tau_181_ increases and changes in cerebral glucose metabolism. In summary, we found that in early Aβ‐accumulators, higher fibrillar Aβ was associated with higher sTREM2 and that sTREM2 mediated the association between earliest PET‐assessed fibrillary Aβ deposition and soluble p‐tau_181_ increases. In contrast, higher sTREM2 was no longer associated with Aβ but paralleled p‐tau_181_ increases in late Aβ‐accumulators, suggesting that sTREM2 is more strongly coupled to the increase in soluble p‐tau_181_ levels once fully developed fibrillary Aβ pathology is present. Higher sTREM2 levels were further associated with FDG‐PET‐assessed glucose hypermetabolism in early Aβ‐accumulators but with glucose hypometabolism in late Aβ‐accumulators. This suggests that increases in glucose metabolism observed in early‐stage AD may reflect Aβ‐related neuroinflammation rather than a compensatory effect (Ashraf *et al*, 2015; Oh *et al*, 2016). Together, our results suggest that a TREM2‐related microglial response is an early element of the amyloid cascade, which is closely associated with earliest p‐tau_181_ increases and metabolic brain changes. This is a critical point when assessing at what stage of the AD continuum we should test drugs targeting TREM2 and microglia. We previously reported protective effects of TREM2‐related microglial responses on attenuated neurodegeneration and symptom progression in later disease stages (Ewers *et al*, 2019, 2020). Further work including longitudinal cognitive data is needed to elucidate if a protective effect also occurs on different pathological brain changes and in earlier stages before there is overt Aβ pathology.

Our first finding showed that the earliest sTREM2‐related microglial response in AD is associated with fibrillar yet subthreshold Aβ increases (i.e., centiloids below 20), and that higher sTREM2 mediated the earliest Aβ‐related increases in soluble p‐tau_181_ levels. In contrast, in participants showing fully developed Aβ pathology as indicated by combined positivity on both CSF and PET (i.e., late Aβ‐accumulators), only associations between fibrillar Aβ (i.e., centiloid) and p‐tau_181_ or between p‐tau_181_ and sTREM2 were found. Notably, we and others (Suarez‐Calvet *et al*, 2019) could not detect any associations between CSF Aβ levels and sTREM2, hence early insoluble but not soluble forms of Aβ might be associated with TREM2‐related microglial responses and subsequent p‐tau increases. Importantly, our results remained consistent when repeating the analysis in a subset of participants with available longitudinal CSF data, showing that sTREM2 increases at baseline predict and mediate subsequent Aβ‐related increases in p‐tau_181_ in early but not in late Aβ‐accumulators. These findings support the view that earliest Aβ fibrillization induces an sTREM2‐related activation of microglia, which is in turn associated with p‐tau_181_ increases, while sTREM2 increases may uncouple from Aβ severity at later stages. Our findings of microglial activation as a mediator of soluble p‐tau_181_ increases is in line with a recent post‐mortem study in older adults showing that activated microglia partly mediated the relationship between Aβ and tau (Casaletto *et al*, 2022). In addition, several preclinical studies showed that activated microglia can enhance tau phosphorylation in animal models of AD and other tauopathies (Bhaskar *et al*, 2010; Lee *et al*, 2014; Maphis *et al*, 2015; Ising *et al*, 2019). Similarly, another study in a rat model of tauopathy could show that animals that are genetically prone to neuroinflammation show stronger neurofibrillary tau pathology compared to animals with less neuroinflammation (Stozicka *et al*, 2010). A further study using brain tissue of AD patients and AD mice could show that microglia phagocytose hyperphosphorylated tau seeds but are incapable of fully neutralizing tau seeding activity and instead release pathological tau seeds into the extracellular space, which can induce a cascade of subsequent tau hyperphosphorylation, misfolding and spread (Hopp *et al*, 2018). Moreover, a large‐scale biomarker study in AD patients with combined amyloid‐PET, tau‐PET, and TSPO‐PET for assessing microglial activation levels could show that elevated microglial activation may promote the aggregation and spreading of fibrillary tau deposits (Pascoal *et al*, 2021). However, other studies show that TREM2 loss of function is associated with a drastically elevated AD risk (Jin *et al*, 2014; Cheng‐Hathaway *et al*, 2018) and with facilitated Aβ‐associated tau seeding in AD mice expressing both Aβ and tau pathology (Leyns *et al*, 2019; Gratuze *et al*, 2021). Further, we reported previously that higher baseline sTREM2 levels in symptomatic AD patients are associated with lower tau‐PET levels several years later (Ewers *et al*, 2020). Conflicting findings of microglial activation on the development of tau pathology may be explained by several factors, including the use of different animal models of AD, which recapitulate different aspects of AD pathophysiology, or using different markers of tau pathology including soluble and fibrillar forms of tau. CSF p‐tau reflects hyperphosphorylated tau in its soluble form, one of the earliest tau‐related changes in AD that is closely associated with Aβ, preceding the formation of intracellular neurofibrillary tau aggregates (Hansson, 2021; Pichet Binette *et al*, 2022). Therefore, microglia may have different effects on p‐tau hyperphosphorylation and aggregation, which will be important to study in greater detail in future studies by combining fluid and PET biomarkers of microglial activation and tau pathology. Future investigations including more comprehensive CSF analyses should compare different CSF p‐tau epitopes (e.g., p‐tau_217_ or p‐tau_231_) to test whether the effect of sTREM2 on p‐tau is consistently observed across different p‐tau species. Further, our study showed that the inclusion of different AD stages can have a drastic impact on the association between sTREM2 levels and downstream AD biomarkers, which may be a key confounder in previous studies. This is also supported by a previous study using an APPPS1‐21 mouse model, showing that a TREM2‐related microglial response can have opposing effects on Aβ pathology, depending on disease stage (Jay *et al*, 2017). Nevertheless, our findings highlight the view that microglia are crucially involved in the amyloid cascade, yet the directionality of effects may be modulated by disease stage and other pathophysiological events. It is important to stress here that microglia exist in a diverse and dynamic states, which respond to diverse physiological and pathological conditions (i.e., Aβ accumulation). The different biomarkers of microglia (e.g., TSPO, CSF sTREM2) may reflect different aspects of the microglial response to the pathology. A current limitation is the lack of other microglial biomarkers that reflect the wide repertoire of microglia states. Therefore, we believe that it is crucial to better understand how microglia and sTREM2 are involved in the molecular progression of tau pathology, from tau hyperphosphorylation and increases in soluble p‐tau to the development and spread of fibrillary tau pathology, in order to evaluate microglia and specifically TREM2‐related pathways as a treatment target.

As a second finding, we report that a TREM2‐related microglial response was associated with increased FDG‐PET‐assessed glucose metabolism in early Aβ‐accumulators, and with decreased glucose metabolism in late Aβ‐accumulators compared to healthy controls. This observation aligns well with previous work, showing that activated microglia consume high levels of glucose (Xiang *et al*, 2021) and should thus be reflected in increased FDG‐PET. The metabolic drop that we observed in later disease might in contrast reflect neurodegenerative processes and neuronal death (Strom *et al*, 2022). Increased glucose metabolism has been previously reported in early AD stages (Ashraf *et al*, 2015; Oh *et al*, 2016) and interpreted as possible compensatory neuronal activity (Ashraf *et al*, 2015), however, proof of an actual advantage of these compensatory mechanisms has been lacking. A match between regional hypermetabolism in early disease stages followed by hypometabolism in later stages has been observed in a previous cross‐sectional study (Oh *et al*, 2016), where cognitively normal older adults with higher levels of Aβ showed glucose hypermetabolism in those regions, which were most susceptible to AD‐related hypometabolism in advanced disease. In addition, in a study including the DIAN cohort, the biggest database of autosomal dominant AD patients, increased FDG‐PET signal was detected ∼25 years before estimated symptom onset in mutation carriers compared to non‐carriers. With advanced disease, hypometabolism was mostly observed in regions that were previously hypermetabolic (Benzinger *et al*, 2013). However, the authors caution the low number of participants (*n* = 11) which were included in the analysis. Importantly, we detected statistically significant associations between a TREM2‐related microglial response and glucose metabolism depending on disease stage. Considering previous work, our findings suggest that different stages of microglial responses and neurodegeneration may induce a non‐linear trajectory of metabolic brain changes in AD. This is also supported by a recent study showing associations between astrogliosis, i.e., another soluble marker of neuroinflammation, and higher FDG‐PET metabolism in earliest AD (Salvado *et al*, 2022). Future studies using more advanced TSPO‐PET for the regional assessment of neuroinflammation should further investigate whether regions affected by high Aβ load show increased signs of neuroinflammation and whether this is coupled by glucose hypermetabolism in earliest AD.

Our findings have important implications for microglial‐related treatment strategies. Previous trials focusing on the reduction of Aβ plaques have mostly failed to significantly reduce cognitive dysfunction, despite some recent positive phase III trials (Knopman *et al*, 2021). Thus, treatments that are directly targeting the pathogenesis of tau, which is much closer linked to neurodegeneration and cognitive decline than Aβ (Bennett *et al*, 2004; Jack *et al*, 2013; Fleisher *et al*, 2015; Ossenkoppele *et al*, 2016; Wang *et al*, 2016; Jack *et al*, 2018), might be more likely to result in a clinical benefit. In the present study, we showed that in earliest Aβ accumulation a TREM2‐related microglial response moderates the association between Aβ and tau, thus, a treatment that targets microglial activation might reduce increases in p‐tau_181_ and therefore, prevent tau aggregates and following neurodegeneration. Here, the time window for an optimal treatment effect will be critical, since our models showed that a TREM2‐related microglial response only mediates p‐tau_181_ increases in early Aβ‐accumulators, while in later disease stages, beneficial effects of activated microglia on cognition could be observed (Ewers *et al*, 2020; Franzmeier *et al*, 2020). Therefore, future studies are highly needed to reveal the ideal time windows for microglial‐related treatment that might target reduction or enhancement of microglia, depending on disease stage.

A clear strength of the present study is its multi‐modal design including CSF biomarkers and PET imaging. When interpreting the data, however, several caveats should be addressed. First, Aβ, p‐tau_181_, and sTREM2 are colinear in the early phase and the mediation analysis was significant both ways, with sTREM2 as mediator for subsequent p‐tau_181_ increases and vice versa. However, the mediation effect was much higher for sTREM2 as mediator than for p‐tau_181_ (*B* = 0.450 compared to *B* = 0.273), suggesting that albeit both, Aβ and p‐tau_181_ may have activating effects on sTREM2, sTREM2 might play a driving role in subsequent p‐tau_181_ elevations. Second, we used CSF Aβ_1‐42_ levels for patients' classification, however, we recognize that a normalization of Aβ using the Aβ_42_/Aβ_40_ ratio would be the preferable measure which should be applied once more data become available in ADNI or other datasets. Further, we cannot ensure that all participants of the early Aβ‐accumulators group will progress to fully developed fibrillar Aβ pathology due to a lack of longitudinal amyloid‐PET data. To minimize the risk of wrong allocation, participants showing an unexpected Aβ biomarker pattern (i.e., CSF−/PET+) or those who already have dementia were excluded from this study (Palmqvist *et al*, 2017). Third, sample sizes were considerably small for early Aβ‐accumulators when including longitudinal CSF sTREM2 (*n* = 21) and CSF p‐tau_181_ (*n* = 20) data, however, results were consistent for both cross‐sectional and longitudinal associations. Further, we could only assess cross‐sectional relationships between sTREM2 and FDG‐PET. Here, it will be critical to assess longitudinal associations between sTREM2 and changes in FDG‐PET once larger longitudinal datasets become available. Fourth, we could not assess p‐tau_181_ by sTREM2 interactions on tau aggregates since tau‐PET data were limited and thus not sufficient to reliably assess relationships with sTREM2 in the current dataset. Here, it would be of high interest to assess the association between tau aggregates, sTREM2, and FDG‐PET. Although we observed group differences (early vs. late Aβ‐accumulators) for the association between sTREM2 and FDG‐PET, the current study cannot disentangle whether sTREM2 plays a moderating role for the association between tau‐PET and FDG‐PET. Nevertheless, we encourage future studies to assess, whether sTREM2 in later disease stages shows attenuating effects on tau accumulation, thereby preventing neuronal death and atrophy which might explain previous findings on protective effects of sTREM2 on neurodegeneration and cognitive decline (Ewers *et al*, 2019). In addition, the current study is to our knowledge the first to assess associations between early versus late‐stage Aβ accumulation, sTREM2, p‐tau, and changes in glucose metabolism. Therefore, replication of our findings in an independent cohort will be an important future endeavor, however, at the time of this study, no comparable dataset was available to us for replication. Finally, for generalization of the results, our analysis should be replicated in ethnically diverse cohorts, since ethnicity has been shown to influence markers of microglial activation (Schindler *et al*, 2021).

## Conclusions

Our findings support disease stage‐dependent effects of microglial responses on AD disease progression. Besides previously reported beneficial effects of a TREM2‐related microglial response in advanced AD, we show that in patients with earliest Aβ abnormalities, microglial activation is associated with increases in p‐tau_181_ and neuroinflammation which is reflected in glucose hypermetabolism. Our findings have important clinical implications as they suggest that targeting enhancement of TREM2‐related microglial responses may have opposing effects on the progression of AD pathophysiology in early versus later stages of AD.

## Materials and Methods

### Participants

We included 402 participants from the Alzheimer's Disease Neuroimaging Initiative (ADNI) with available CSF Aβ_1‐42_, p‐tau_181_, and sTREM2, ^18^F‐florbetapir/^18^F‐florbetaben amyloid‐PET, FDG‐PET as well as demographics (sex, age, education) and clinical status. Baseline CSF and PET data had to be obtained within a time window of 6 months. Clinical status was classified by ADNI investigators as cognitively normal (CN; Mini Mental State Examination [MMSE] ≥ 24, Clinical Dementia Rating [CDR] = 0, non‐depressed), mild cognitive impairment (MCI; MMSE ≥ 24, CDR = 0.5, objective memory‐impairment on education‐adjusted Wechsler Memory Scale II, preserved activities of daily living) or dementia (MMSE = 20–26, CDR ≥ 0.5, NINCDS/ADRDA criteria for probable AD). ADNI inclusion/exclusion criteria can be found at https://adni.loni.usc.edu/wp‐content/uploads/2010/09/ADNI_GeneralProceduresManual.pdf. Participants with dementia were excluded from this study, since we followed a previous approach of disease stage stratification (Palmqvist *et al*, 2017), excluding later stages of AD. To determine disease stage, participants were stratified into early or late Aβ‐accumulators based on their Aβ CSF and amyloid‐PET status (Palmqvist *et al*, 2017). Aβ CSF positivity was determined as CSF Aβ_1‐42_ < 976.6 pg/ml (Suarez‐Calvet *et al*, 2019) and amyloid‐PET positivity was determined as ^8^F‐florbetapir > 1.11 SUVR (Landau *et al*, 2012) or ^18^F‐florbetaben PET > 1.08 SUVR (Royse *et al*, 2021). Participants were then grouped as early Aβ‐accumulators (Aβ CSF+/PET−; CN/MCI *n* = 30/40) and as late Aβ‐accumulators (CSF+/PET+; CN/MCI *n* = 41/160). Participants which were classified as Aβ CSF−/PET+ were excluded (*n* = 39). In addition, Aβ CSF−/PET− participants were included as a healthy reference group. One participant had abnormal Aβ CSF levels (~4,000 pg/ml) and was thus excluded, resulting in 131 controls comprising of CN participants only. A subset of participants had available longitudinal p‐tau_181_ (early/late/controls *n* = 20/75/35) and sTREM2 (early/late/controls *n* = 21/75/35) assessments, based on which we calculated annual p‐tau_181_ and sTREM2 change rates. Ethical approval was obtained by ADNI investigators, and all study participants provided written informed consent.

### Ethics approval and consent to participate

Ethics approval was obtained by the ADNI investigators from the local ethical committees of all involved sites. The study was conducted in accordance with the Declaration of Helsinki and all study participants provided written informed consent. All work complied with ethical regulations for work with human participants.

### CSF biomarkers

Aβ_1‐42_ and p‐tau_181_ levels were assessed by the ADNI biomarker core team at the University of Pennsylvania. An electrochemiluminiscence immunoassays Elecsys on a fully automated Elecsys cobas e 601 instrument and a single lot of reagents for each biomarker were used. For the assessment of sTREM2, a previously described ELISA approach was applied (Kleinberger *et al*, 2014; Suarez‐Calvet *et al*, 2016; Ewers *et al*, 2019). sTREM2 data are provided in the ADNI_HAASS_WASHU_LAB.csv file available in the ADNI database (variable “MSD_STREM2CORRECTED”). A detailed description of the methods is found online (https://ida.loni.usc.edu).

### MRI and PET acquisition and preprocessing

3T structural MRI was obtained by ADNI employing T1‐weighted MPRAGE sequences using unified scanning protocols (http://adni.loni.usc.edu/methods/mri‐tool/mri‐analysis/). Amyloid‐PET was recorded 50–70 min after ^18^F‐florbetapir injection in 4 × 5 min frames or 90–110 min after ^18^F‐florbetaben injection in 4 × 5 min frames. FDG‐PET was recorded 30–60 min after ^18^F‐flurodeoxyglucose injection in 6 × 5 min frames. To obtain mean images, recorded time frames were motion corrected and averaged (see also http://adni.loni.usc.edu/methods/pet‐analysis‐method/pet‐analysis/). Using the Advanced Normalization Tools (ANTs; Avants *et al*, 2011) high‐dimensional warping algorithm, nonlinear spatial normalization parameters to Montreal Neurological Institute (MNI) space were estimated based on structural skull‐stripped T1‐weighted images. Amyloid‐PET and FDG‐PET images were then co‐registered to native‐space T1‐weighted images and subsequently normalized to MNI space by applying the ANTs‐derived normalization parameters. Amyloid‐PET SUVRs were intensity normalized to the whole cerebellum and FDG‐PET SUVRs to the pons. To harmonize between both amyloid‐PET tracers, global amyloid‐PET SUVRs across ^18^F‐florbetapir and ^18^F‐florbetaben were transformed to centiloid using equations provided by ADNI (Klunk *et al*, 2015).

### Statistical analyses

All statistical analyses were computed using R statistical software version 4.0.2 (http://www.R‐project.org; R Core Team, 2021).

Baseline characteristics between groups were compared using ANOVAs for continuous and chi‐squared tests for categorical data.

To test whether a TREM2‐related microglial response is associated with soluble p‐tau_181_ increases in early Aβ‐accumulators, we first assessed cross‐sectional disease stage‐dependent (i.e., early vs. late Aβ‐accumulators) associations between centiloid and CSF sTREM2, and between centiloid and CSF p‐tau_181_, using linear regressions. Here, we used sTREM2 or p‐tau_181_ as the dependent variable, and centiloid as the independent variable. To assess longitudinal disease stage‐dependent associations, we first calculated slope estimates for annual change rates of CSF sTREM2 and CSF p‐tau_181_ for those participants with available longitudinal sTREM2 and p‐tau_181_ data. Here, linear mixed models were fitted using sTREM2 or p‐tau_181_ as dependent variable and time (i.e., years from baseline) as independent variable, adjusting for subject‐specific random slope and intercept. Subsequently, we performed linear regressions using annual change rates of sTREM2 or p‐tau_181_ as the dependent variable and baseline centiloid as the independent variable. In addition, analyses were repeated using CSF Aβ instead of centiloid for testing associations between soluble Aβ and sTREM2 or p‐tau_181_. The regression models were controlled for age, sex, education, and clinical status (i.e., CN or MCI). The models testing the control group were not controlled for clinical status since the group only comprised of CN participants. Finally, bootstrapped mediation analyses using 1,000 iterations were applied using the mediation package in R (https://cran.r‐project.org/web/packages/mediation/mediation.pdf) for assessing cross‐sectional and longitudinal associations, using centiloid as the predictor variable, p‐tau_181_ (i.e., cross‐sectional) or p‐tau_181_ change rate (i.e., longitudinal) as the dependent variable, and sTREM2 as the mediator. Subsequently, we tested the reverse associations (i.e., p‐tau_181_ as the mediator of centiloid's effect on sTREM2). All mediation analyses were controlled for age, sex, education, and clinical status. Lastly, we combined early and late Aβ‐accumulators and tested for an sTREM2 by group interaction on cross‐sectional and longitudinal p‐tau_181_ levels, controlling for age, sex, education, and clinical status.

In the next step, we assessed whether a TREM2‐related microglial response manifests in glucose hypermetabolism in early AD stages when neurodegeneration is typically not yet apparent (i.e., Aβ CSF+/PET−) versus glucose hypometabolism in later disease stage (i.e., Aβ CSF+/PET+). To this end, we first calculated FDG‐PET *z*‐scores by referencing FDG‐PET signals to 131 CN Aβ− controls. We then tested for an sTREM2 by group (i.e., Aβ CSF+/PET− vs. Aβ CSF+/PET+) interaction on FDG‐PET signal in a meta‐ROI that typically captures AD‐related glucose metabolism changes (Landau *et al*, 2011). For *post hoc* exploration, we repeated the analysis in a brain wide manner across 200 ROIs that cover the entire neocortex (Schaefer *et al*, 2018), in order to map the pattern of microglial activation effects on glucose hyper‐ versus hypometabolism depending on disease stage. To that end, we computed linear regressions for the associations between sTREM2 and each of the 200 FDG‐PET ROIs. Note that the latter analysis was only exploratory to assess the general pattern between sTREM2 and FDG‐PET in early versus late Aβ‐accumulators and thus not corrected for multiple comparisons. To test whether early/late Aβ‐accumulators differ regarding sTREM2‐related glucose metabolism, *T*‐values of the association between sTREM2 and FDG‐PET were compared between groups using *t*‐tests. For non‐parametric comparison, we determined 95% confidence intervals (CI) for the 200 *T*‐values for early and late Aβ‐accumulators. The models were controlled for age, sex, education, and clinical status. Finally, the main analyses were repeated including APOE4 status as a covariate to assess whether APOE4 influences the effect of sTREM2 on p‐tau_181_ or glucose metabolism. Subjects were classified as APOE4 risk allele carriers when at least one ε4 allele was detected.

## Author contributions

**Davina Biel:** Conceptualization; data curation; formal analysis; investigation; writing – original draft; writing – review and editing. **Marc Suárez‐Calvet:** Writing – review and editing. **Paul Hager:** Data curation; writing – review and editing. **Anna Rubinski:** Writing – review and editing. **Anna Dewenter:** Writing – review and editing. **Anna Steward:** Writing – review and editing. **Sebastian Roemer:** Writing – review and editing. **Michael Ewers:** Writing – review and editing. **Christian Haass:** Data curation; writing – review and editing. **Matthias Brendel:** Conceptualization; writing – review and editing. **Nicolai Franzmeier:** Conceptualization; data curation; formal analysis; investigation; writing – original draft; writing – review and editing. Open Access funding enabled and organized by Projekt DEAL.

## Disclosure and competing interests statement

MSC has served as a consultant and at advisory boards for Roche Diagnostics International Ltd and has given lectures in symposia sponsored by Roche Diagnostics, S.L.U and Roche Farma, S.A. NF has served as a consultant for Merck Sharp & Dohme. The authors declare no further competing interests.

## Supporting information



Expanded View Figures PDF

Table EV1

Table EV2

PDF+

Source Data for Figure 1

Source Data for Figure 2

## Data Availability

All data used in this manuscript are available from the ADNI database (adni.loni.usc.edu) upon registration and compliance with the data use agreement. The data that support the findings of this study are available on reasonable request from the corresponding author. Source data of the figures of this manuscript are available online on the BioStudies database (https://www.ebi.ac.uk/biostudies/studies/S‐BSST954).
