# Supplementary material for: sTREM2 is associated with amyloid‐related p‐tau increases and glucose hypermetabolism in Alzheimer's disease
Source: EMBO Mol Med. 2023 Jan 9;15(2):e16987. doi: 10.15252/emmm.202216987 (PMC9906389; doi:10.15252/emmm.202216987)
Supplement: Supplementary file 1 — Expanded View Figures PDF [file EMMM-15-e16987-s003.pdf]

Expanded View Figures

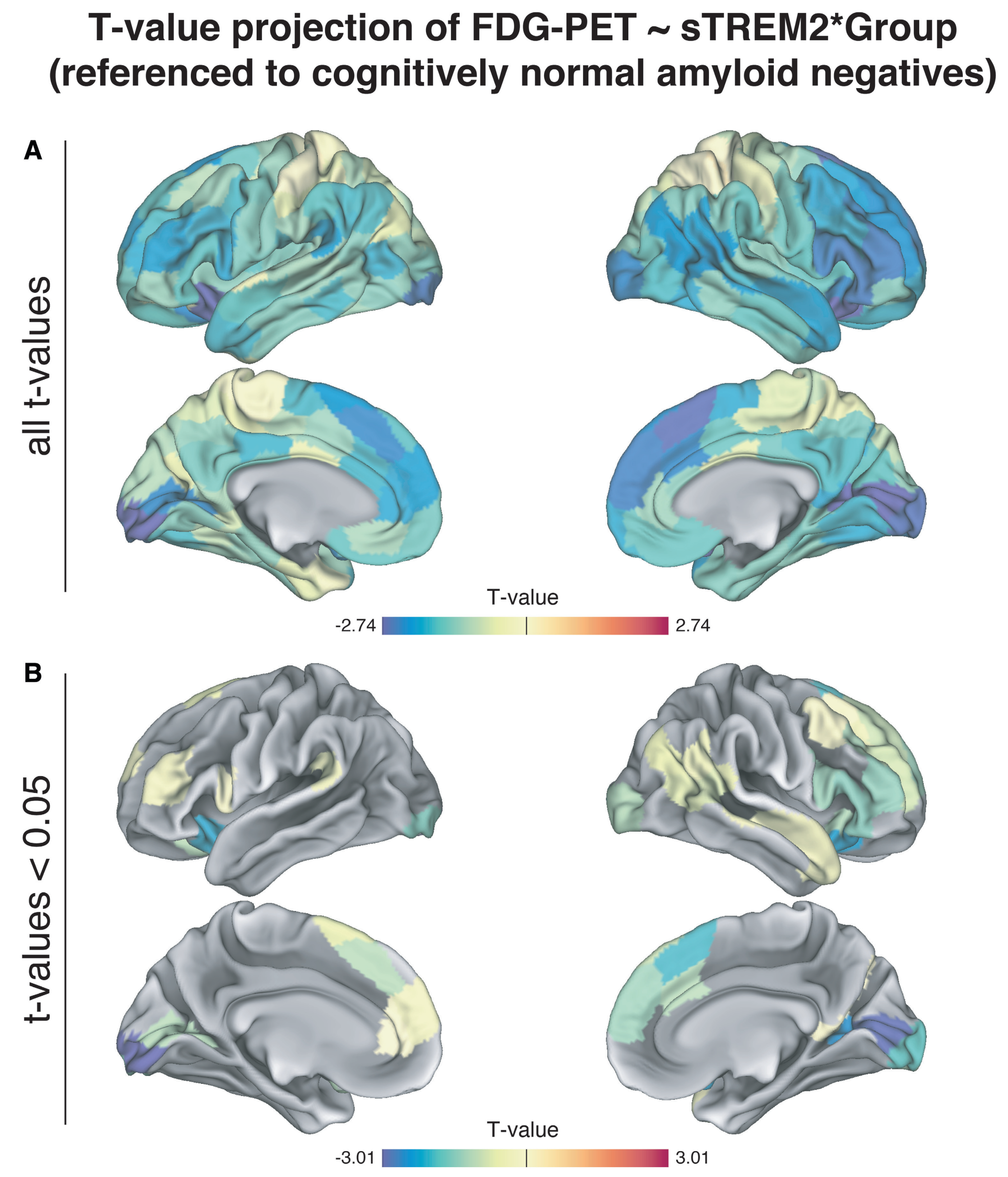

Figure EV1. *T*-value projection of the CSF sTREM2 by group (early Aβ-accumulators [Aβ CSF+/PET−] vs. late Aβ-accumulators [Aβ CSF+/PET+]) interaction on FDG-PET.

A Projection of all *T*-values across 200 ROIs of the Schaefer brain atlas.
B Projection of *T*-values that are below a *P*-value of 0.05.

Data information: FDG-PET *z*-scores were derived by referencing FDG-PET SUVRs to cognitively normal controls (i.e., $n = 131$; Aβ CSF−/PET−). The models are controlled for age, sex, education, and clinical status.

© 2023 The Authors
