# Supplementary material for: sTREM2 is associated with amyloid‐related p‐tau increases and glucose hypermetabolism in Alzheimer's disease
Source: EMBO Mol Med. 2023 Jan 9;15(2):e16987. doi: 10.15252/emmm.202216987 (PMC9906389; doi:10.15252/emmm.202216987)
Supplement: Supplementary file 2 — Table EV1 [file EMMM-15-e16987-s001.docx]

**Table EV1.** Regression analyses assessing associations between amyloid-PET (i.e. centiloid), sTREM2, and p-tau_181_. The table displays standardized beta-estimates (β), T-values, and p-values. The regression models are controlled for age, sex, education, clinical status, and APOE4.

|  | **Cross-sectional** | | | **Longitudinal** | | |
| --- | --- | --- | --- | --- | --- | --- |
|  | β | T | p | β | T | p |
|  | *Early Aβ-accumulators (Aβ CSF+/PET−)* | | | | | |
| p-tau_181_ ~ centiloid | 0.234 | 2.050 | 0.045 | 0.486 | 2.719 | 0.018 |
| sTREM2 ~ centiloid | 0.247 | 2.183 | 0.033 | 0.484 | 3.318 | 0.005 |
| p-tau_181_ ~ sTREM2 | 0.566 | 5.462 | <0.001 | 0.877 | 5.419 | <0.001 |
|  | *Late Aβ-accumulators (Aβ CSF+/PET+)* | | | | | |
| p-tau_181_ ~ centiloid | 0.201 | 2.951 | 0.004 | 0.138 | 1.156 | 0.252 |
| sTREM2 ~ centiloid | 0.046 | 0.663 | 0.508 | 0.040 | 0.360 | 0.720 |
| p-tau_181_ ~ sTREM2 | 0.457 | 7.239 | <0.001 | 0.451 | 3.805 | <0.001 |
