# Supplementary material for: sTREM2 is associated with amyloid‐related p‐tau increases and glucose hypermetabolism in Alzheimer's disease
Source: EMBO Mol Med. 2023 Jan 9;15(2):e16987. doi: 10.15252/emmm.202216987 (PMC9906389; doi:10.15252/emmm.202216987)
Supplement: Supplementary file 3 — Table EV2 [file EMMM-15-e16987-s005.docx]

**Table EV2.** Mediation analyses with amyloid-PET (i.e. centiloid) as predictor, sTREM2 as mediator, and p-tau_181_ as dependent variable. The table displays the average causal mediation effect (ACME), the average direct effect (ADE), Beta-estimates (B), 95% confidence intervals (CI), and p-values. The models are controlled for age, sex, education, clinical status, and APOE4.

|  | **Cross-sectional** | | | **Longitudinal** | | |
| --- | --- | --- | --- | --- | --- | --- |
|  | B | 95%CI | p-value | B | 95%CI | p-value |
|  | *Early Aβ-accumulators (Aβ CSF+/PET−)* | | | | | |
| ACME | 0.125 | –0.006 - 0.250 | 0.058 | 0.374 | 0.077 - 0.710 | 0.010 |
| ADE | 0.102 | −0.090 - 0.300 | 0.322 | 0.100 | −0.218 - 0.440 | 0.546 |
|  | *Late Aβ-accumulators (Aβ CSF+/PET+)* | | | | | |
| ACME | 0.020 | −0.047 - 0.080 | 0.488 | 0.0002 | −0.105 - 0.110 | 0.960 |
| ADE | 0.177 | 0.058 - 0.300 | 0.006 | 0.135 | −0.082 - 0.360 | 0.230 |
