# Supplementary material for: sTREM2 is associated with amyloid‐related p‐tau increases and glucose hypermetabolism in Alzheimer's disease
Source: EMBO Mol Med. 2023 Jan 9;15(2):e16987. doi: 10.15252/emmm.202216987 (PMC9906389; doi:10.15252/emmm.202216987)
Supplement: Supplementary file 7 [file EMMM-15-e16987-s002.pdf]

EMBO Molecular Medicine

sTREM2 is associated with amyloid-related p-tau increases and glucose hypermetabolism in Alzheimer's

Davina Biel, Marc Suárez-Calvet, Paul Hager, Anna Rubinski, Anna Dewenter, Anna Steward, Sebastian Roemer, Michael Ewers, Christian Haass, Matthias Brendel, and Nicolai Franzmeier
DOI: 10.15252/emmm.202216987

Corresponding author: Nicolai Franzmeier (Nicolai.Franzmeier@med.uni-muenchen.de)

Review Timeline:

Submission Date:	4th Oct 22
Editorial Decision:	3rd Nov 22
Revision Received:	25th Nov 22
Editorial Decision:	20th Dec 22
Revision Received:	21st Dec 22
Accepted:	22nd Dec 22

Editor: Jingyi Hou

Transaction Report:

(Note: With the exception of the correction of typographical or spelling errors that could be a source of ambiguity, letters and reports are not edited. Depending on transfer agreements, referee reports obtained elsewhere may or may not be included in this compilation. Referee reports are anonymous unless the Referee chooses to sign their reports.)

3rd Nov 2022

Dear Dr. Franzmeier,

Thank you again for submitting your work to EMBO Molecular Medicine. We have heard back from three referees who agreed to evaluate your manuscript. As you will see from the reports below, the referees acknowledge the potential interest of the study. However, they raise a series of concerns, which we would ask you to address in a major revision of the manuscript.

I think that the referees' recommendations are relatively straightforward, so there is no need to reiterate their comments. All issues raised by the referees need to be satisfactorily addressed. As you may already know, our editorial policy allows in principle a single round of major revision so it is essential to provide responses to the reviewers' comments that are as complete as possible. Please feel free to contact me in case you would like to discuss in further detail any of the issues raised by the reviewers.

We would welcome the submission of a revised version within three months for further consideration. EMBO Molecular Medicine has a "scooping protection" policy, whereby similar findings that are published by others during review or revision are not a criterion for rejection. Should you decide to submit a revised version, I do ask that you get in touch after three months if you have not completed it to update us on the status.

We are aware that many laboratories cannot function at full efficiency during the current COVID-19/SARS-CoV-2 pandemic and have therefore extended our "scooping protection policy" to cover the period required for a full revision to address the experimental issues. Please let me know should you need additional time, and also if you see a paper with related content published elsewhere.

Please read below for important editorial formatting and consult our author's guidelines for proper formatting of your revised article for EMBO Molecular Medicine.

I look forward to receiving your revised manuscript.

Use this link to login to the manuscript system and submit your revision: https://embomolmed.msubmit.net/cgi-bin/main.plex

Sincerely,
Jingyi

Jingyi Hou
Editor
EMBO Molecular Medicine

***** Reviewer's comments *****

Referee #1 (Remarks for Author):

In this study, Biel and colleagues have demonstrated that in people with early amyloid accumulation, that is those with abnormal CSF Aβ42 levels but normal Aβ-PET scans (Aβ CSF+/PET-), higher baseline Aβ-PET centiloid were associated with increased baseline and longitudinal levels of CSF soluble Triggering Receptor Expressed on Myeloid Cell 2 (sTREM2) and p-tau181. Furthermore, the authors report that in Aβ CSF+/PET-, the associations between Aβ-PET and cross-sectional as well as longitudinal CSF p-tau181 were mediated by sTREM2. In late Aβ-accumulators (Aβ CSF+/PET+), no associations were seen between Aβ-PET and sTREM2, whereas there was a positive association between baseline CSF levels of sTREM2 and p-tau181. Lastly, the author found opposite associations between sTREM2 and FDG-PET in early vs. late Aβ-accumulators; higher CSF levels of sTREM2 were associated with glucose hypermetabolism and hypometabolism in Aβ CSF+/PET- and Aβ CSF+/PET-, respectively. The manuscript is generally well-written and the results are clearly presented. The topic of the study is of high interest given that understanding of the relationship between TREM2 and Alzheimer's disease (AD)-related Aβ and tau pathologies is critical for design of therapeutic strategies targeting TREM2 to promote protective functions of microglia in AD. However, the lack of longitudinal imaging data is a limitation of the study and even though longitudinal CSF sTREM2 and p-tau181 data were available the samples size was small particularly in the early Aβ-accumulator group. This is my main concern especially considering a recent paper from DIAN that have highlighted the importance of longitudinal data when investigating the relationship between sTREM2 and AD pathological processes. Other issues that need to be addressed:

1. CFF Aβ42 was used to classify study participants as early or late Aβ-accumulators. However, the ratio of Aβ42 to Aβ40 would be a better measure because it is less affected by non-AD inter-individual differences in total Aβ levels.
2. In statistical analysis, adjustment for multiples comparisons should be applied.
3. Since there is a significant sTREM2 by group interaction on FDG-PET, it would be of interest to display the association for both early or late Aβ-accumulator groups.
4. It is unclear whether sTREM2 levels were associated with cross-sectional or longitudinal p-tau181 levels in early Aβ-accumulators.
5. Were there any associations between longitudinal changes in sTREM2 levels and longitudinal changes in p-tau181 or CSF Aβ levels.
6. The authors could consider focusing the introduction and the discussion specifically on the sTREM2 studies rather than on the role of microglia in AD.
7. In the discussion, parts of the first paragraph and the beginning of the second paragraphs are repetitive.

Referee #2 (Comments on Novelty/Model System for Author):

The statical analysis seems sometimes a bit of data stretch and does not follow the most commonly used methods. I would like to see if the results will remain doing more straightforward analysis of the FDG results.

Referee #2 (Remarks for Author):

Biel and colleagues studied 402 ADNI participants stratified into early Aβ accumulators (CSF+/PET−) and late Aβ accumulators (CSF+/PET+) based on assumptions that the CSF Aβ abnormality occurs before PET Aβ. They reported that in the early Aβ accumulators, Aβ was associated with TREM2 and p-tau, and TREM2 mediated the association of Aβ and p-tau. Higher TREM2 was associated with glucose hypermetabolism. On the other hand, in late accumulators, Aβ was not associated with TREM2, but TREM2 was associated with p-tau and glucose hypometabolism.

This is an interesting manuscript on an important topic which is the association between Aβ, tau phosphorylation, and glial markers. Results showing that TREM2 plays a role in the link between Aβ deposition and tau hyperphosphorylation are very relevant to the field and may have important implications for the biological definitions of the disease.

I have some concerns with the analysis

- Is the significant interaction term reported to FDG also significant for p-tau?

- The manuscript's title suggests that TREM2 mediates amyloid-associated glucose hypermetabolism. It is not clear from the analyses the presence of mediation in glucose metabolism. Was there significant mediation with FDG?

- The analysis in Figure 2B seems a bit of a stretch. The authors made associations in 2 groups anchored in controls and then compared T values of these associations from various regions. Are any of that T values significant after multiple comparisons in the first place? or the authors are comparing a bunch of non-significant t-value ? I would like to see the actual significant T-map after multiple comparison correction in each one of the groups.
Can the authors compare t-values with the method they did statically speaking?

- The link between the results obtained with p-tau and FDG is not very clear and seems sometimes disconnected.

Referee #3 (Comments on Novelty/Model System for Author):

These studies are excellently conducted and statistically analyzed and present incremental, yet important insight into the relationship of sTREM2 as a surrogate for microglial activation and the progression of AD.

Referee #3 (Remarks for Author):

In this study Biel and coworkers describe the relationship between amyloid, sTREM2, and pTau 181 in early stage of amyloid and late stages of amyloid pathology. The authors find that higher levels of amyloid by PET were associated with higher sTREM2 and p-tau181, where as in late accumulators higher sTREM2 was associated with higher p-tau181 with no association with amyloid PET. Interestingly, sTREM2 was associated wither higher glucose metabolism in early accumulators, but lower in late accumulators. The relationship between metabolism, amyloid, tau, and neuroinflammation is important to understanding the disease process and possible timing of therapeutics targeting microglia. Additional analyses could increase the power of the study.

Major critques:
1.) Given the authors' previous work on the relationship between sTREM2 and APOE4 it would be interesting to include APOE4 as a covariate in the study to assess whether the effects of sTREM2 on ptau-181 and glucose metabolism were influenced by APOE genotype
2.) It would be interesting to extend the comparisons with other CSF p-tau epitopes (ie p-tau 217 or p-tau 231) if available to see if sTREM2 influences those phosphorylations as well.

We thank the editor and the reviewer's for their encouraging feedback on our manuscript. Below please find our replies including a description of all changes that were made to the manuscript according to the comments raised by the reviewer's. We hope that we have sufficiently addressed all concerns and believe that the manuscript has been improved and strengthened during the review process.

Please note that we merged two summary paragraphs in the result section (p. 11).

Referee #1 (Remarks for Author):

In this study, Biel and colleagues have demonstrated that in people with early amyloid accumulation, that is those with abnormal CSF Aβ42 levels but normal Aβ-PET scans (Aβ CSF+/PET-), higher baseline Aβ-PET centiloid were associated with increased baseline and longitudinal levels of CSF soluble Triggering Receptor Expressed on Myeloid Cell 2 (sTREM2) and p-tau181. Furthermore, the authors report that in Aβ CSF+/PET-, the associations between Aβ-PET and cross-sectional as well as longitudinal CSF p-tau181 were mediated by sTREM2. In late Aβ-accumulators (Aβ CSF+/PET+), no associations were seen between Aβ-PET and sTREM2, whereas there was a positive association between baseline CSF levels of sTREM2 and p-tau181. Lastly, the author found opposite associations between sTREM2 and FDG-PET in early vs. late Aβ-accumulators; higher CSF levels of sTREM2 were associated with glucose hypermetabolism and hypometabolism in Aβ CSF+/PET- and Aβ CSF+/PET-, respectively. The manuscript is generally well-written and the results are clearly presented. The topic of the study is of high interest given that understanding of the relationship between TREM2 and Alzheimer's disease (AD)-related Aβ and tau pathologies is critical for design of therapeutic strategies targeting TREM2 to promote protective functions of microglia in AD. However, the lack of longitudinal imaging data is a limitation of the study and even though longitudinal CSF sTREM2 and p-tau181 data were available the samples size was small particularly in the early Aβ-accumulator group. This is my main concern especially considering a recent paper from DIAN that have highlighted the importance of longitudinal data when investigating the relationship between sTREM2 and AD pathological processes. Other issues that need to be addressed:

> Reply:
> We thank the reviewer for the thoughtful comments on our manuscript. We agree that the lack of longitudinal imaging data is a shortcoming of this study and further acknowledge this limitation in the discussion of the manuscript.
> p. 18: *"Third, sample sizes were considerably small for early Aβ-accumulators when including longitudinal CSF sTREM2 (n=21) and CSF p-tau$_{181}$ (n=20) data, however, results were consistent for both cross-sectional and longitudinal associations. Further, we could only assess cross-sectional relationships between sTREM2 and FDG-PET. Here, it will be critical to assess longitudinal associations between sTREM2 and changes in FDG-PET once larger longitudinal datasets become available."*

1. CFF Aβ42 was used to classify study participants as early or late Aβ-accumulators. However, the ratio of Aβ42 to Aβ40 would be a better measure because it is less affected by non-AD inter-individual differences in total Aβ levels.

> Reply:
> We agree that a normalization of CSF Aβ using the $A\beta_{42}/A\beta_{40}$ ratio would be the preferable measure. Unfortunately, $A\beta_{42}/A\beta_{40}$ ratio data are only available on ADNI since 2019, and most of the sTREM2 assessments have been obtained before. Therefore, we could not generate sufficient data using this ratio in the current sample. We added this point in the limitations section.

p. 17/18: "*Second, we used CSF A$\beta_{1\text{–}42}$ levels for patients' classification, however, we recognize that a normalization of Aβ using the Aβ_{42}/Aβ_{40} ratio would be the preferable measure which should be applied once more data become available in ADNI or other datasets.*"

2. In statistical analysis, adjustment for multiples comparisons should be applied.

Reply:
In our first analysis testing the association between Centiloid, CSF sTREM2 and CSF p-tau$_{181}$, different samples (early vs. late Aβ-accumulators) and different datasets (cross vs. longitudinal) were used testing different hypotheses. Hence, no correction for multiple comparisons was statistically needed, given that different hypotheses were addressed. For the assessment of the relationship between CSF sTREM2 and FDG-PET, the interaction term reached significance (Fig.2A) and the surface rendering of t-values was an additional exploratory step (Fig.2B). We would like to point out that the latter analysis should underpin a different association pattern between sTREM2 and FDG-PET in early vs. late accumulators and not locate to specific ROIs that drive the relationship that was observed for the FDG-PET meta-ROI. We added a section in the methods to clarify the exploratory character of this analysis.
p. 8*: "For post-hoc exploration, we repeated the analysis in a brain wide manner across 200 ROIs that cover the entire neocortex (…). Note that the latter analysis was only exploratory to assess the general pattern between sTREM2 and FDG-PET in early vs. late Aβ-accumulators and thus not corrected for multiple comparisons."*

3. Since there is a significant sTREM2 by group interaction on FDG-PET, it would be of interest to display the association for both early or late Aβ-accumulator groups.

Reply:
We would like to highlight that we have already displayed the association between sTREM2 and FDG-PET for both groups separately in Fig.2B. Therefore, we are assuming that the reviewer refers to a surface rendering of the interaction effect across the pooled sample (lm[each ROI ~ sTREM2 * Group + age + sex + education + diagnosis]). We added a respective figure in the Extended View.
p. 13: *"The brain surface projection of the T-values of the sTREM2 by group interaction on FDG-PET is made available in the Expanded View (**Fig. EV1**).".* This figure will be made available online.

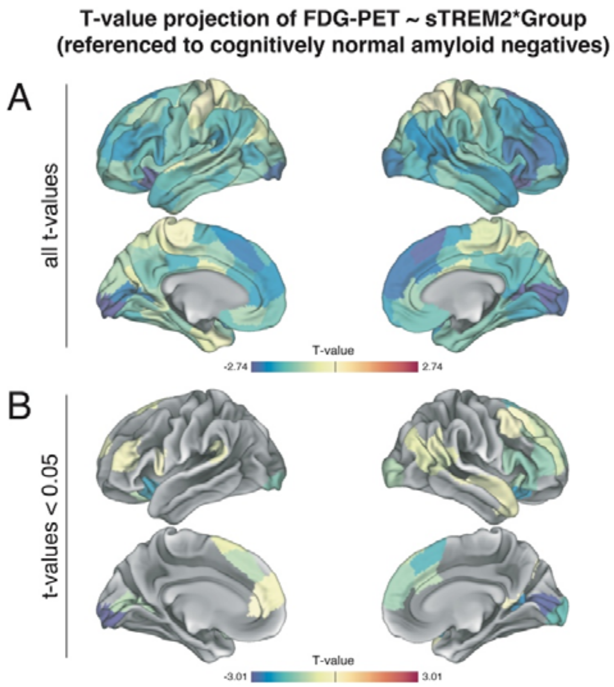

Figure EV1. T-value projection of the CSF sTREM2 by group (early Aβ-accumulators [Aβ CSF+/PET–] vs. late Aβ-accumulators [Aβ CSF+/PET+]) interaction on FDG-PET. FDG-PET z-scores were derived by referencing FDG-PET SUVRs to cognitively normal controls (i.e. *n*=131; Aβ CSF–/PET–). The models are controlled for age, sex, education, and clinical status. **A)** Projection of all T-values across 200 ROIs of the Schaefer brain atlas and **B)** projection of T-values that fall below a p-value of 0.05.

4. It is unclear whether sTREM2 levels were associated with cross-sectional or longitudinal p-tau181 levels in early Aβ-accumulators.

Reply:
We thank the reviewer for pointing out that the longitudinal association between sTREM2 and p-tau_{181} was missing for early Aβ-accumulators. The cross-sectional association between sTREM2 and p-tau_{181} was already included in the manuscript: *"Further, higher sTREM2 levels were associated with higher p-tau_{181} levels (β=0.587, T=5.425, p<0.001)."*

We added the missing longitudinal analysis.
p. 10: *"We obtained congruent results using longitudinal CSF data, (…). In addition, higher baseline sTREM2 levels were associated with faster subsequent change rates in p-tau_{181} (β=0.938, T=6.286, p<0.001)."*

5. Were there any associations between longitudinal changes in sTREM2 levels and longitudinal changes in p-tau181 or CSF Aβ levels.

Reply:
To answer the reviewers question we tested associations between longitudinal changes in sTREM2 and longitudinal changes in p-tau_{181} or CSF $A\beta_{1-42}$. We found significant associations between changes in sTREM2 and changes in p-tau_{181} in both groups (early Aβ-accumulators [n=31]: β=0.571, T=2.901, p=0.008; late Aβ-accumulators [n=111]: β=0.293, T=2.967, p=0.004). We did not observe associations between changes in sTREM2 and changes in CSF $A\beta_{1-42}$ (early Aβ-accumulators [n=31]: β=-0.121, T=-0.537, p=0.596; late Aβ-accumulators

[n=111]: β=0.147, T=1.435, p=0.154). The models were corrected for age, sex, education, and clinical status.

Since we needed to generate an additional dataset including longitudinal CSF $A\beta_{1-42}$ for this analysis and are unsure whether this analysis will bring added value to the manuscript, we decided not to include it in the final version. However, it will be available online in the rebuttal in case the manuscript is accepted for publication.

6. The authors could consider focusing the introduction and the discussion specifically on the sTREM2 studies rather than on the role of microglia in AD.

Reply:

Several studies we referred to in the introduction or discussion were already using sTREM2 as a proxy for microglial activation. As in the papers themselves, we were using the expression "microglial activation" as a synonym for sTREM2. However, we clarified now in our manuscript which cited studies used sTREM2.

p. 3: *"Here, activation of microglia, the brains innate immune system, may play a key role in modulating these initial events in the amyloid cascade (10). The Triggering Receptor Expressed on Myeloid Cell 2 (TREM2) regulates the change of microglia from a homeostatic state to a disease associated state (11, 12), and is a well-established in vivo proxy for microglial activation in AD (13-17). Yet, previous studies have yielded conflicting findings on a detrimental or protective role of microglial activation or TREM2-related microglial responses in AD."*

p. 3: *"Similarly, studies in sporadic AD patients found that a TREM2-related microglial response is strongly correlated with soluble p-tau but not with $A\beta$ levels (20) and that a TREM2-related microglial response may promote the development of aggregated tau pathology in AD, as measured via tau-PET (10, 21)."*

p. 4: "On the contrary, in symptomatic sporadic AD patients and patients with autosomal dominantly inherited AD, a higher TREM2-related microglial response has been associated with attenuated cognitive decline, amyloid accumulation and neurodegeneration (13, 28, 29)."

p. 4: *"Similarly, an animal model has shown that TREM2 loss of function is associated with increased $A\beta$ seeding, (…)"*

p. 4: *"In the present study, we used CSF soluble TREM2 (sTREM2) (…)"*

Although we used sTREM2 as a proxy of microglial activation, we think that referring to studies using different assessments of microglial activation (i.e. TSPO-PET, post-mortem, etc.) is important to gain a holistic picture of the involvement of microglia in the pathogenesis of AD. We already included a paragraph addressing this topic in the discussion.

p. 16: *"The different biomarkers of microglia (e.g. TSPO, CSF sTREM2) may reflect different aspects of the microglial response to the pathology. A current limitation is the lack of other microglial biomarkers that reflect the wide repertoire of microglia states. Therefore, we believe that it is crucial to better understand how microglia and sTREM2 are involved in the molecular progression of tau pathology, from tau hyperphosphorylation and increases in soluble p-tau to the development and spread of fibrillary tau pathology, in order to evaluate microglia and specifically TREM2-related pathways as a treatment target."*

7. In the discussion, parts of the first paragraph and the beginning of the second paragraphs are repetitive.

Reply:
We agree with the reviewer and shortened the beginning of the second paragraph.
p. 14: *"Our first finding showed that the earliest sTREM2-related microglial response in AD is associated with fibrillar yet subthreshold $A\beta$ increases (i.e. centiloids below 20), and that higher sTREM2 mediated the earliest $A\beta$-related increases in soluble p-tau_{181} levels. In contrast, in participants showing fully developed $A\beta$ pathology as indicated by combined positivity on both CSF and PET (i.e. late $A\beta$-accumulators), only associations between fibrillar $A\beta$ (i.e. centiloid) and p-tau_{181} or between p-tau_{181} and sTREM2 were found."*

Referee #2 (Comments on Novelty/Model System for Author):

The statical analysis seems sometimes a bit of data stretch and does not follow the most commonly used methods. I would like to see if the results will remain doing more straightforward analysis of the FDG results.

Referee #2 (Remarks for Author):

Biel and colleagues studied 402 ADNI participants stratified into early Aβ accumulators (CSF+/PET−) and late Aβ accumulators (CSF+/PET+) based on assumptions that the CSF Aβ abnormality occurs before PET Aβ. They reported that in the early Aβ accumulators, Aβ was associated with TREM2 and p-tau, and TREM2 mediated the association of Aβ and p-tau. Higher TREM2 was associated with glucose hypermetabolism. On the other hand, in late accumulators, Aβ was not associated with TREM2, but TREM2 was associated with p-tau and glucose hypometabolism.

This is an interesting manuscript on an important topic which is the association between Aβ, tau phosphorylation, and glial markers. Results showing that TREM2 plays a role in the link between Aβ deposition and tau hyperphosphorylation are very relevant to the field and may have important implications for the biological definitions of the disease.

Reply:
We appreciate the reviewers' reasoned comments and suggestions for improving our manuscript. We hope we have sufficiently addressed the remaining concerns.

I have some concerns with the analysis

- Is the significant interaction term reported to FDG also significant for p-tau?

Reply:
This is an interesting question, however, the interaction term for an sTREM2 by group interaction on p-tau levels did not reach significance (lm[p-tau_{181}~ sTREM2 *Group + age + sex + education + clinical status]. We included this analysis in the methods and results.
p. 8: *"Lastly, we combined early and late $A\beta$-accumulators and tested for an sTREM2 by group interaction on cross-sectional and longitudinal p-tau_{181} levels, controlling for age, sex, education, and clinical status."*
p. 12: *"No sTREM2 by group interaction on CSF p-tau_{181} levels were found when pooling early and late $A\beta$-accumulators (cross-sectional interaction term: β=0.022, T=0.145, p=0.885; longitudinal interaction term: β=–0.091, T=–0.345, p=0.731)."*

This suggests that sTREM2 is associated with higher p-tau levels across the AD continuum, but that glucose metabolism is differently associated with sTREM2. This is in line with previous work, showing that activated microglia consume a majority of the FDG-PET tracer (Xiang et al., Sci Transl Med, 2021), which may result in the observed hypermetabolism when neurodegeneration and resulting hypometabolic brain changes are not yet apparent. Please note also, that we have discussed this in the paper (p. 16+17).

- The manuscript's title suggests that TREM2 mediates amyloid-associated glucose hypermetabolism. It is not clear from the analyses the presence of mediation in glucose metabolism. Was there significant mediation with FDG?

Reply:
We agree with the reviewer that we did not explicitly test a mediation effect for the association between sTREM2 and amyloid-associated glucose hypermetabolism. Therefore, we appreciate the comment that the title can be misleading and changed it into *"sTREM2 is associated with amyloid-related p-tau increases and glucose hypermetabolism in Alzheimer's"*

- The analysis in Figure 2B seems a bit of a stretch. The authors made associations in 2 groups anchored in controls and then compared T values of these associations from various regions. Are any of that T values significant after multiple comparisons in the first place? or the authors are comparing a bunch of non-significant t-value ? I would like to see the actual significant T-map after multiple comparison correction in each one of the groups.
Can the authors compare t-values with the method they did statically speaking?

Reply:
For the assessment of the relationship between CSF sTREM2 and FDG-PET, our main analyses focused on the interaction term of group (i.e. early vs. late Aβ-accumulators) by sTREM2 on the FDG-PET meta-ROI (i.e. a summary ROI of AD-vulnerable brain regions) reached significance (Fig.2A). This was the significant main analysis on which we base our conclusion that sTREM2 relates to increased metabolism in early Aβ-accumulators vs. decreased metabolism in late Aβ-accumulators. The surface rendering of T-values was an additional exploratory step (Fig.2B), in which we aimed to examine whether the association between sTREM2 and hypo/hypermetabolism was generally apparent across the brain or restricted to particular brain regions. We would like to point out that the latter analysis should underpin a different overall association pattern of sTREM2 with FDG-PET between early and late Aβ-accumulators and not locate specific ROIs with a significant interaction effect. Note also, that we tested main effects of sTREM2 and not interactions with group here. We added a section in the methods to clarify the exploratory character of this analysis. Therefore, the T-values can rather be seen as an effect size measure that indicates the directionality of an association between sTREM2 and FDG-PET in the early vs. late accumulators.
p. 8*: "For post-hoc exploration, we repeated the analysis in a brain wide manner across 200 ROIs that cover the entire neocortex (…). Note that the latter analysis was only exploratory to assess the general pattern between sTREM2 and FDG-PET in early vs. late Aβ-accumulators and thus not corrected for multiple comparisons."*

In addition to the t-test we added a non-parametrical comparison using confidence intervals (CI), which provide non parametric support that associations between sTREM2 and FDG-PET show an overall opposing directionality in early vs. late Aβ-accumulators.

p. 8+9: *"For non-parametric comparison, we determined 95% confidence intervals (CI) for the 200 T-values for early and late Aβ-accumulators."*
p. 13: *"In addition, 95% CIs of the 200 T-values did not overlap (early Aβ-accumulators: CI 1.086-1.249; late Aβ-accumulators: CI –1.302-–1.098) (…)."*
Please note also that reviewer 1 raised a similar concern and asked us to repeat the sTREM2 by group interaction analyses on a group level, and show the full T-map, which is shown in our response to comment 3 by reviewer 1.

- The link between the results obtained with p-tau and FDG is not very clear and seems sometimes disconnected.

Reply:
We understand the reviewers concern and added an exploratory section in the limitation. The rationale of including FDG-PET was that we have previously shown that p-tau precedes tau aggregates (Pichet Binette et al., Nat Comm, 2022), which in turn ensue neurodegeneration. Since not sufficient tau-PET data were available, we could not directly assess the effect of sTREM2 on the association between p-tau and tau-PET or tau-PET and neurodegeneration. Therefore, we only assessed the link between sTREM2 (which we showed to be associated with subsequent p-tau increases) and neurodegeneration (i.e. FDG-PET). However, including tau-PET to the equation will be an important step for future studies.
p. 18: *"Fourth, we could not assess p-tau$_{181}$ by sTREM2 interactions on tau aggregates since tau-PET data were limited and thus not sufficient to reliably assess relationships with sTREM2 in the current dataset. Here, it would be of interest to assess the association between tau aggregates, sTREM2, and FDG-PET. Although we observed group differences (early vs. late Aβ-accumulators) for the association between sTREM2 and FDG-PET, the current study cannot disentangle whether sTREM2 plays a moderating role for the association between tau-PET and FDG-PET. (…)."*

Referee #3 (Comments on Novelty/Model System for Author):

These studies are excellently conducted and statistically analyzed and present incremental, yet important insight into the relationship of sTREM2 as a surrogate for microglial activation and the progression of AD.

Reply:
We thank the reviewer for the overall positive feedback on our manuscript and the helpful suggestions for improvements.

Referee #3 (Remarks for Author):

In this study Biel and coworkers describe the relationship between amyloid, sTREM2, and pTau 181 in early stage of amyloid and late stages of amyloid pathology. The authors find that higher levels of amyloid by PET were associated with higher sTREM2 and p-tau181, where as in late accumulators higher sTREM2 was associated with higher p-tau181 with no association with amyloid PET. Interestingly, sTREM2 was associated wither higher glucose metabolism in early accumulators, but lower in late accumulators. The relationship between metabolism, amyloid, tau, and neuroinflammation is important to understanding the disease process and possible timing of therapeutics targeting microglia. Additional analyses could increase the power of the study.

Major critques:

1.) Given the authors' previous work on the relationship between sTREM2 and APOE4 it would be interesting to include APOE4 as a covariate in the study to assess whether the effects of sTREM2 on ptau-181 and glucose metabolism were influenced by APOE genotype

Reply:

We repeated the main analyses including APOE4 status and added the results in the Expanded View.

p. 9: *"Finally, the main analyses were repeated including APOE4 status as a covariate to assess whether APOE4 influences the effect of sTREM2 on p-tau$_{181}$ or glucose metabolism. Subjects were classified as APOE4 risk allele carriers when at least one ε4 allele was detected."*

p. 11: *"All analyses remained consistent when including APOE4 genotype as a covariate (except for the cross-sectional ACME in early Aβ-accumulators, which only reached borderline significance (p=0.058; Table EV1+EV2))."*

p. 13: *"The analyses remained consistent when including APOE4 genotype as a covariate (β=–0.379, T=–1.972, p=0.050)."*

Table EV1. Regression analyses assessing associations between amyloid-PET (i.e. centiloid), sTREM2, and p-tau$_{181}$. The table displays standardized beta-estimates (β), T-values, and p-values. The regression models are controlled for age, sex, education, clinical status, and APOE4.

	**Cross-sectional**			**Longitudinal**		
	β	T	p	β	T	p
	Early Aβ-accumulators (Aβ CSF+/PET–)					
p-tau$_{181}$ ~ centiloid	0.234	2.050	0.045	0.486	2.719	0.018
sTREM2 ~ centiloid	0.247	2.183	0.033	0.484	3.318	0.005
p-tau$_{181}$ ~ sTREM2	0.566	5.462	<0.001	0.877	5.419	<0.001
	Late Aβ-accumulators (Aβ CSF+/PET+)					
p-tau$_{181}$ ~ centiloid	0.201	2.951	0.004	0.138	1.156	0.252
sTREM2 ~ centiloid	0.046	0.663	0.508	0.040	0.360	0.720
p-tau$_{181}$ ~ sTREM2	0.457	7.239	<0.001	0.451	3.805	<0.001

Table EV2. Mediation analyses with amyloid-PET (i.e. centiloid) as predictor, sTREM2 as mediator, and p-tau$_{181}$ as dependent variable. The table displays the average causal mediation effect (ACME), the average direct effect (ADE), Beta-estimates (B), 95% confidence intervals (CI), and p-values. The models are controlled for age, sex, education, clinical status, and APOE4.

	**Cross-sectional**			**Longitudinal**		
	B	95%CI	p-value	B	95%CI	p-value
	Early Aβ-accumulators (Aβ CSF+/PET–)					
ACME	0.125	–0.006 - 0.250	0.058	0.374	0.077 - 0.710	0.010
ADE	0.102	–0.090 - 0.300	0.322	0.100	–0.218 - 0.440	0.546
	Late Aβ-accumulators (Aβ CSF+/PET+)					
ACME	0.020	–0.047 - 0.080	0.488	0.0002	–0.105 - 0.110	0.960
ADE	0.177	0.058 - 0.300	0.006	0.135	–0.082 - 0.360	0.230

2.) It would be interesting to extend the comparisons with other CSF p-tau epitopes (ie p-tau 217 or p-tau 231) if available to see if sTREM2 influences those phosphorylations as well.

Reply:

We agree with the reviewer that other CSF p-tau epitopes would be interesting to look at, unfortunately, at the current stage, only p-tau$_{181}$ is available in ADNI. We added this outlook in the discussion.

p. 15+16*: "Future investigations including more comprehensive CSF analyses should compare different CSF p-tau epitopes (e.g. p-tau$_{217}$ or p-tau$_{231}$) to test whether the effect of sTREM2 on p-tau is consistently observed across different p-tau species."*

20th Dec 2022

Dear Dr. Franzmeier,

Thank you for the submission of your revised manuscript to EMBO Molecular Medicine. We have received the enclosed reports from the three referees who agreed to re-assess it. As you will see, while Reviewers #2 and #3 are now supportive, Reviewer #1 still raised some concerns about the generalizability of the findings and requested replication in an independent cohort. We would ask you to discuss this raised point as potential limitations and future directions in writing.

On a more editorial level:
- Since the final manuscript only has two main figures, we suggest that you change the manuscript type from "Article" to "Reports" in the next submission.

- Please reduce the keyword number to five.

- Reference format: Please list up to 10 co-authors of a paper before adding et al. to the reference list.

- Move The Paper Explained to the main manuscript file.

- Materials and Methods should be placed after the Discussion. Figures should be removed from the manuscript file. The list of abbreviations should be removed. The ethics approval should be in the Materials and Methods section.

- We updated our journal's competing interests policy in January 2022 and request authors to consider both actual and perceived competing interests. Please review the policy https://www.embopress.org/competing-interests and update your competing interests if necessary.
Please use the heading "Disclosure statement and competing interests".

- Funding should be merged with Acknowledgement. Funding information entered in the online submission system should match that listed in the manuscript text. German Research Foundation (DFG, INST 409/193-1 FUGG) was missing from the online submission system.

- Please provide a striking image or visual abstract to illustrate your article as a PNG file 550 px wide x 300-600 px high.

- Our data editors have seen the manuscript, and they have made some comments and suggestions that need to be addressed (see attached). I have also slightly modified the Data availability section. Please send back a revised version (in track change mode), as we will need to go through the changes.

- As part of the EMBO Publications transparent editorial process initiative (see our Editorial at http://embomolmed.embopress.org/content/2/9/329), EMBO Molecular Medicine will publish online a Review Process File (RPF) to accompany accepted manuscripts.

In the event of acceptance, this file will be published in conjunction with your paper and will include the anonymous referee reports, your point-by-point response, and all pertinent correspondence relating to the manuscript. Let us know whether you disagree with this and if you want to remove or keep any figures from it prior to publication.

Please note that the Authors checklist will be published at the end of the RPF.

I look forward to reading a new revised version of your manuscript as soon as possible.

Sincerely,
Jingyi

Jingyi Hou
Editor
EMBO Molecular Medicine

***** Reviewer's comments *****

Referee #1 (Remarks for Author):

I do not believe the authors fully addressed my concerns. Acknowledgment of the lack of longitudinal data, small sample size and the use of CSF Aβ42 to define early accumulators as limitations of the study is not, in my opinion, enough. Given these caveats I think replication in an independent cohort is needed which will also ensure the generalizability of the findings.

Referee #2 (Remarks for Author):

The authors answered my concerns satisfactorily

Referee #3 (Comments on Novelty/Model System for Author):

I reiterate that this is an excellently conducted study.

Referee #3 (Remarks for Author):

The authors have answered my queries well and I support publication.

Referee #1 (Remarks for Author):

I do not believe the authors fully addressed my concerns. Acknowledgment of the lack of longitudinal data, small sample size and the use of CSF Aβ42 to define early accumulators as limitations of the study is not, in my opinion, enough. Given these caveats I think replication in an independent cohort is needed which will also ensure the generalizability of the findings.

Response: We thank the reviewer for again reviewing our manuscript and we appreciate the concern that a validation cohort would be desirable to replicate our results. However, there is no large-enough cohort currently available to us that is characterized by both CSF and PET amyloid measures, sTREM2 and FDG-PET including longitudinal data. Therefore, full replication of our results will be an important future endeavor which we will follow once large enough data become available from collaborators that are willing to share their data for this purpose. To address the reviewers' concern, we have added a statement to the limitations section of the manuscript in the discussion, clearly arguing that replication will be important, but that the requested replication sample is currently not available to us. We hope that these final edits sufficiently address the reviewers' concern, especially given that we have submitted a detailed point-by-point response to all previously raised concerns which the reviewer raised.

Referee #2 (Remarks for Author):
The authors answered my concerns satisfactorily

Response: We thank the reviewer for these encouraging remarks.

Referee #3 (Comments on Novelty/Model System for Author):
I reiterate that this is an excellently conducted study.
Referee #3 (Remarks for Author):
The authors have answered my queries well and I support publication.

Response: We thank the reviewer for the positive evaluation and for these encouraging comments.

22nd Dec 2022

Dear Dr. Franzmeier,

I am pleased to inform you that your manuscript is accepted for publication and is now being sent to our publisher to be included in the next available issue of EMBO Molecular Medicine.

We would like to remind you that as part of the EMBO Publications transparent editorial process initiative, EMBO Molecular Medicine will publish a Review Process File online to accompany accepted manuscripts. If you do NOT want the file to be published or would like to exclude figures, please immediately inform the editorial office via e-mail.

Please read below for additional IMPORTANT information regarding your article, its publication and the production process.

Congratulations on your interesting work,

Jingyi

Jingyi Hou
Editor
EMBO Molecular Medicine

EMBO Press Author Checklist

Corresponding Author Name: Nicolai Franzmeier, PhD	
Journal Submitted to: EMBO Molecular Medicine	
Manuscript Number: EMM-2022-16987	

USEFUL LINKS FOR COMPLETING THIS FORM
The EMBO Journal - Author Guidelines
EMBO Reports - Author Guidelines
Molecular Systems Biology - Author Guidelines
EMBO Molecular Medicine - Author Guidelines

Reporting Checklist for Life Science Articles (updated January 2022)
This checklist is adapted from Materials Design Analysis Reporting (MDAR) Checklist for Authors. MDAR establishes a minimum set of requirements in transparent reporting in the life sciences (see Statement of Task: 10.31222/osf.io/9sm4x). Please follow the journal's guidelines in preparing your manuscript.
Please note that a copy of this checklist will be published alongside your article.

Abridged guidelines for figures
1. Data
The data shown in figures should satisfy the following conditions:
- the data were obtained and processed according to the field's best practice and are presented to reflect the results of the experiments in an accurate and unbiased manner.
- ideally, figure panels should include only measurements that are directly comparable to each other and obtained with the same assay.
- plots include clearly labeled error bars for independent experiments and sample sizes. Unless justified, error bars should not be shown for technical replicates.
- if n<5, the individual data points from each experiment should be plotted. Any statistical test employed should be justified.
- Source Data should be included to report the data underlying figures according to the guidelines set out in the authorship guidelines on Data Presentation.

2. Captions
Each figure caption should contain the following information, for each panel where they are relevant:
- a specification of the experimental system investigated (eg cell line, species name).
- the assay(s) and method(s) used to carry out the reported observations and measurements.
- an explicit mention of the biological and chemical entity(ies) that are being measured.
- an explicit mention of the biological and chemical entity(ies) that are altered/varied/perturbed in a controlled manner.
- the exact sample size (n) for each experimental group/condition, given as a number, not a range;
- a description of the sample collection allowing the reader to understand whether the samples represent technical or biological replicates (including how many animals, litters, cultures, etc.).
- a statement of how many times the experiment shown was independently replicated in the laboratory.
- definitions of statistical methods and measures:
  - common tests, such as t-test (please specify whether paired vs. unpaired), simple χ2 tests, Wilcoxon and Mann-Whitney tests, can be unambiguously identified by name only, but more complex techniques should be described in the methods section;
  - are tests one-sided or two-sided?
  - are there adjustments for multiple comparisons?
  - exact statistical test results, e.g., P values = x but not P values < x;
  - definition of 'center values' as median or average;
  - definition of error bars as s.d. or s.e.m.

Please complete ALL of the questions below.
Select "Not Applicable" only when the requested information is not relevant for your study.

Materials

Newly Created Materials	Information included in the manuscript?	In which section is the information available? (Reagents and Tools Table, Materials and Methods, Figures, Data Availability Section)
New materials and reagents need to be available; do any restrictions apply?	Not Applicable	

Antibodies	Information included in the manuscript?	In which section is the information available? (Reagents and Tools Table, Materials and Methods, Figures, Data Availability Section)
For **antibodies** provide the following information:<br>- Commercial antibodies: RRID (if possible) or supplier name, catalogue number and or/clone number<br>- Non-commercial: RRID or citation	Not Applicable	

DNA and RNA sequences	Information included in the manuscript?	In which section is the information available? (Reagents and Tools Table, Materials and Methods, Figures, Data Availability Section)
**Short novel DNA or RNA including primers, probes**: provide the sequences.	Not Applicable	

Cell materials	Information included in the manuscript?	In which section is the information available? (Reagents and Tools Table, Materials and Methods, Figures, Data Availability Section)
**Cell lines:** Provide species information, strain. Provide accession number in repository **OR** supplier name, catalog number, clone number, and/**OR** RRID.	Not Applicable	
**Primary cultures:** Provide species, strain, sex of origin, genetic modification status.	Not Applicable	
Report if the cell lines were recently **authenticated** (e.g., by STR profiling) and tested for mycoplasma contamination.	Not Applicable	

Experimental animals	Information included in the manuscript?	In which section is the information available? (Reagents and Tools Table, Materials and Methods, Figures, Data Availability Section)
**Laboratory animals or Model organisms:** Provide species, strain, sex, age, genetic modification status. Provide accession number in repository **OR** supplier name, catalog number, clone number, **OR** RRID.	Not Applicable	
**Animal observed in or captured from the field:** Provide species, sex, and age where possible.	Not Applicable	
Please detail **housing and husbandry conditions**.	Not Applicable	

Plants and microbes	Information included in the manuscript?	In which section is the information available? (Reagents and Tools Table, Materials and Methods, Figures, Data Availability Section)
**Plants:** provide species and strain, ecotype and cultivar where relevant, unique accession number if available, and source (including location for collected wild specimens).	Not Applicable	
**Microbes:** provide species and strain, unique accession number if available, and source.	Not Applicable	

Human research participants	Information included in the manuscript?	In which section is the information available? (Reagents and Tools Table, Materials and Methods, Figures, Data Availability Section)
If collected and within the bounds of privacy constraints report on age, sex and gender or ethnicity for all study participants.	Yes	Table 1

Core facilities	Information included in the manuscript?	In which section is the information available? (Reagents and Tools Table, Materials and Methods, Figures, Data Availability Section)
If your work benefited from core facilities, was their service mentioned in the acknowledgments section?	Yes	Acknowledgements

Design

Study protocol	Information included in the manuscript?	In which section is the information available? (Reagents and Tools Table, Materials and Methods, Figures, Data Availability Section)
If study protocol has been **pre-registered, provide DOI in the manuscript**. For clinical trials, provide the trial registration number **OR** cite DOI.	Not Applicable	
Report the **clinical trial registration number** (at ClinicalTrials.gov or equivalent), where applicable.	Not Applicable	

Laboratory protocol	Information included in the manuscript?	In which section is the information available? (Reagents and Tools Table, Materials and Methods, Figures, Data Availability Section)
Provide DOI OR other citation details if **external detailed step-by-step protocols** are available.	Not Applicable	

Experimental study design and statistics	Information included in the manuscript?	In which section is the information available? (Reagents and Tools Table, Materials and Methods, Figures, Data Availability Section)
Include a statement about **sample size** estimate even if no statistical methods were used.	Yes	Materials and Methods - Participants
Were any steps taken to minimize the effects of subjective bias when allocating animals/samples to treatment (e.g. **randomization procedure**)? If yes, have they been described?	Not Applicable	
Include a statement about **blinding** even if no blinding was done.	Not Applicable	
Describe **inclusion/exclusion criteria** if samples or animals were excluded from the analysis. Were the criteria pre-established?<br><br>If sample or data points were omitted from analysis, report if this was due to attrition or intentional exclusion and provide justification.	Yes	Materials and Methods - Participants
For every figure, are **statistical tests** justified as appropriate? Do the data meet the assumptions of the tests (e.g., normal distribution)? Describe any methods used to assess it. Is there an estimate of variation within each group of data? Is the variance similar between the groups that are being statistically compared?	Yes	Materials and Methods - Statistics

Sample definition and in-laboratory replication	Information included in the manuscript?	In which section is the information available? (Reagents and Tools Table, Materials and Methods, Figures, Data Availability Section)
In the figure legends: state number of times the experiment was **replicated** in laboratory.	Not Applicable	
In the figure legends: define whether data describe **technical or biological replicates**.	Not Applicable	

Ethics

Ethics	Information included in the manuscript?	In which section is the information available? (Reagents and Tools Table, Materials and Methods, Figures, Data Availability Section)
Studies involving **human participants**: State details of **authority granting ethics approval** (IRB or equivalent committee(s), provide reference number for approval.	Yes	Declarations - Ethics approval and consent to participate
Studies involving **human participants**: Include a statement confirming that **informed consent** was obtained from all subjects and that the experiments conformed to the principles set out in the WMA Declaration of Helsinki and the Department of Health and Human Services Belmont Report.	Yes	Declarations - Ethics approval and consent to participate
Studies involving **human participants:** For publication of **patient photos**, include a statement confirming that consent to publish was obtained.	Not Applicable	
Studies involving experimental **animals**: State details of **authority granting ethics approva**l (IRB or equivalent committee(s), provide reference number for approval. Include a statement of compliance with ethical regulations.	Not Applicable	
Studies involving **specimen and field samples:** State if relevant **permits** obtained, provide details of authority approving study; if none were required, explain why.	Not Applicable	

Dual Use Research of Concern (DURC)	Information included in the manuscript?	In which section is the information available? (Reagents and Tools Table, Materials and Methods, Figures, Data Availability Section)
Could your study fall under dual use research restrictions? Please check biosecurity documents and list of **select agents and toxins** (CDC): https://www.selectagents.gov/sat/list.htm	Not Applicable	
If you used a select agent, is the security level of the lab appropriate and reported in the manuscript?	Not Applicable	
If a study is subject to dual use research of concern regulations, is the name of the **authority granting approval and reference number** for the regulatory approval provided in the manuscript?	Not Applicable	

Reporting

The MDAR framework recommends adoption of discipline-specific guidelines, established and endorsed through community initiatives. Journals have their own policy about requiring specific guidelines and recommendations to complement MDAR.

Adherence to community standards	Information included in the manuscript?	In which section is the information available? (Reagents and Tools Table, Materials and Methods, Figures, Data Availability Section)
State if relevant guidelines or checklists (e.g., **ICMJE, MIBBI, ARRIVE, PRISMA**) have been followed or provided.	Not Applicable	
For **tumor marker prognostic studies,** we recommend that you follow the **REMARK** reporting guidelines (see link list at top right). See author guidelines, under 'Reporting Guidelines'. Please confirm you have followed these guidelines.	Not Applicable	
For **phase II and III randomized controlled trials**, please refer to the **CONSORT** flow diagram (see link list at top right) and submit the CONSORT checklist (see link list at top right) with your submission. See author guidelines, under 'Reporting Guidelines'. Please confirm you have submitted this list.	Not Applicable	

Data Availability

Data availability	Information included in the manuscript?	In which section is the information available? (Reagents and Tools Table, Materials and Methods, Figures, Data Availability Section)
Have **primary datasets** been deposited according to the journal's guidelines (see 'Data Deposition' section) and the respective accession numbers provided in the Data Availability Section?	Yes	Data availability section
Were **human clinical and genomic datasets** deposited in a public access-controlled repository in accordance to ethical obligations to the patients and to the applicable consent agreement?	Not Applicable	
Are **computational models** that are central and integral to a study available without restrictions in a machine-readable form? Were the relevant accession numbers or links provided?	Not Applicable	
If publicly available data were reused, provide the respective **data citations in the reference list**.	Not Applicable	
